# Levetiracetam restores GABAergic function and network homeostasis following *Vps35* deficiency in excitatory neurons

**DOI:** 10.1186/s40035-026-00585-x

**Published:** 2026-10-14

**Authors:** Zhi-Peng Liu, Daehoon Lee, Zhibing Tan, Anika Wu, Lin Mei, Wen-Cheng Xiong

**Affiliations:** 1 Department of Neurosciences, Case Western Reserve University, School of Medicine, Cleveland, OH 44106 USA; 2 State Key Laboratory of Transvascular Implantation Devices, The Second Affiliated Hospital, Zhejiang University, Hangzhou, 310009 China; 3 Chinese Institute of Medical Research, Capital Medical University, Beijing, 100069 China

**Keywords:** Alzheimer’s disease, VPS35, Retromer, Levetiracetam, GABAergic transmission, E/I balance

## Abstract

**Background:**

Alzheimer’s disease (AD) and related dementias (ADRDs) remain incurable, partly due to their complex and poorly understood pathogenic mechanisms. Loss of vacuolar protein sorting-associated protein 35 (VPS35), a core component of the retromer complex, has been implicated in AD and ADRDs. While *Vps35* deficiency is known to increase amyloidogenesis by regulating its cargo proteins, such as APP and BACE1, the precise mechanism by which VPS35 dysfunction leads to cognitive impairment remains elusive. While neuronal VPS35 regulates trafficking and functions of multiple synaptic membrane proteins, such as postsynaptic glutamatergic and GABAnergic receptors and pre-synaptic vesicle protein SV2A, and Levetiracetam (LEV), an antiepileptic drug that improves cognition, interacts with the SV2A, exactly how LEV enhances cognition is still a matter of debate.

**Methods:**

Given the abundant expression of VPS35 in pyramidal excitatory neurons, we generated a conditional knockout mouse model with *Vps35* deletion from postnatal glutamatergic excitatory neurons (*Vps35*^*CaMKIIα*^) and characterized its phenotypes using a combination of behavioral assessments, and electrophysiological, biochemical, and immunohiotological analyses in the hippocampal CA1 region. A targeted pharmacological screen was conducted to evaluate the therapeutic potential of LEV.

**Results:**

*Vps35* deletion did not disrupt the gross brain development but led to progressive cognitive decline beginning at three months of age. Electrophysiological analyses revealed earlier-onset hippocampal CA1 hyperexcitability, marked by depolarized resting membrane potentials and reduced Kir_2.1_ expression. However, restoring Kir_2.1_ to suppress hyperexcitability failed to rescue cognitive deficits. Instead, cognitive impairment was associated with weakened GABAergic transmission and disruption of the excitatory/inhibitory balance in the Schaffer collateral–CA1 circuit. Notably, LEV treatment diminished these deficits in mutant mice. Intriguingly, LEV increased the levels of retromer component, VPS26A, and multiple pre- and postsynaptic GABAergic proteins, in addition to SV2A, all of which were reduced in *Vps35*-deficient CA1 neurons.

**Conclusions:**

These findings identify VPS35 in excitatory neurons as a critical factor for inhibitory synapse maintenance and network homeostasis. This study also highlights LEV as a potential therapeutic strategy for ADRDs through the preservation of GABAergic synapses.

**Supplementary Information:**

The online version contains supplementary material available at https://doi.org/10.1186/s40035-026-00585-x.

## Background

Impairment in cognitive function is a hallmark of dementia, which includes Alzheimer’s disease (AD) and related dementias (ADRDs) such as frontotemporal dementia (FTD), vascular dementia (VaD), Down syndrome-associated dementia (DS), and Lewy body dementia (LBD) [1–5]. Each dementia subtype is defined by distinct neuropathological features, such as amyloid plaques and phosphorylated tau tangles in AD, phosphorylated TDP-43 or tau aggregates in FTD, and α-synuclein-positive Lewy bodies in LBD [6–10]. However, the pathogenesis of dementias involves a range of genetic and environmental risk factors, with disease progression driven by complex neurodegenerative processes that ultimately lead to dysfunction or loss of neurons, particularly pyramidal neurons (PyNs) in the hippocampus and cortex [11, 12]. Thus, understanding how specific genetic factors contribute to PyN dysfunction and cognitive decline is critical for uncovering disease mechanisms and informing therapeutic development.

The balance between excitatory and inhibitory neurotransmission (E/I balance) is essential for normal brain function. Disruption of this balance represents a pathological shift from physiological conditions and is implicated in several neurological disorders, including seizures, AD, and ADRDs [13–18]. Patients with AD/ADRDs often exhibit neuronal hyperactivity and epileptiform activity, both associated with and contributing to the E/I imbalance [19, 20]. These abnormalities, such as irregular oscillations, network hypersynchrony, and epileptiform discharges, are commonly detected by functional magnetic resonance imaging and electroencephalography (EEG) [19, 21–23]. Recent studies using fluorescence deconvolution tomography and synaptic membrane micro-transplantation have reported elevated synaptic E/I ratios in the post-mortem parietal cortex of individuals with familial AD [13]. Similar E/I imbalance and circuit hyperactivity have been observed in the hippocampus and cortex of AD/ADRD transgenic mouse models, as well as in human induced pluripotent stem cell-derived neuronal cultures and cerebral organoids [24–26]. These alterations often precede or coincide with cognitive decline, raising critical questions about the role of hyperactivity and E/I imbalance in dementia pathophysiology.

Interestingly, several anti-epileptic drugs (AEDs), such as levetiracetam (LEV), lamotrigine (LAMO), and valproic acid (VPA), have been reported with beneficial effects in dementia patients [27–30]. While LEV effectively suppresses hyperexcitability driven by excessive synaptic release, it is not uniformly effective across all forms of network E/I imbalance, particularly oscillation-driven absence seizures [31, 32]. AD is primarily associated with focal seizure and generalized tonic–clonic seizure (GTCS) rather than absence seizures [33, 34]. These observations raise additional questions how AD is associated with focal seizure and/or GTCS; whether and how AEDs, such as LEV, suppress focal and/or GTCS, and thus the development of AD and/or ADRD.

While each ADRD subtype is associated with distinct genetic risk factors, *VPS35* (vacuolar protein sorting-associated protein 35) is emerging as a common player across multiple neurodegenerative diseases. VPS35 is a core component of the retromer complex, which includes VPS35, VPS26, and VPS29, and mediates the trafficking of transmembrane proteins from endosomes to the trans-Golgi network or plasma membrane [35, 36]. Decreased VPS35 or retromer expression has been reported in key brain regions affected by various disorders: the hippocampus in AD, frontal cortex in FTD, substantia nigra in Parkinson’s disease (PD), and specific areas in DS [37–42]. Additionally, pathogenic *VPS35* mutations have been identified in autosomal-dominant PD [41, 43–45], suggesting a broader role for VPS35 dysfunction in neurodegeneration.

Although regional VPS35 deficits are documented across disorders, *Vps35* heterozygous mice exhibit neuropathological features relevant to both PD, such as α-synuclein accumulation and dopaminergic neuron loss, and AD, including elevated Aβ levels, impaired glutamatergic signaling, and accelerated AD progression [38, 46]. Several VPS35/retromer cargo proteins are directly linked to AD and ADRDs. For instance, amyloid precursor protein (APP), TREM2, and sortilin-related receptor (SorLA) are known AD risk factors and VPS35 cargos [47–50]. Sortilin1, another VPS35 cargo, is a receptor for progranulin and implicated in FTD pathogenesis [51]. These findings suggest that VPS35 deficiency may disrupt cargo trafficking across different brain cell types or regions, contributing to diverse neurodegenerative processes. However, the mechanism by which VPS35 dysfunction accelerates specific ADRD subtypes and cognitive decline remains elusive.

Our previous studies have shown that VPS35 is highly expressed in the developing and young adult PyNs of the hippocampus and neocortex, regions particularly vulnerable in AD/ADRDs [46, 52–54]. Selective deletion of *Vps35* in developing PyNs, as in *Vps35*^*Neurod6−Cre*^ mice, impairs neuronal terminal differentiation and induces brain pathology, including TDP-43 accumulation, disrupted autophagy-lysosomal processes, and neonatal lethality [40]. In contrast, postnatal deletion of *Vps35* in young adult PyNs (*Vps35*^*CaMKIIa*^ mice at 2 months of age) does not produce these early developmental deficits [40, 54], suggesting a distinct developmental role of VPS35 in regulating cargo proteins and neuronal functions, potentially contributing to distinct neurodegenerative outcomes.

To further investigate adult neuronal VPS35/retromer’s function, we further characterized *Vps35*^*CaMKIIa*^ mice via electrophysiological, behavioral, neuropharmacological, and immunohistochemical assays. These mutant mice exhibit progressive AD- and ADRD-related phenotypes, including cognitive behavioral deficits, impaired hippocampal long-term potentiation (LTP), CA1 neuronal hyperexcitability, and E/I imbalance. Further studies yield three key conclusions. First, suppressing CA1 neuronal hyperexcitability by modulating Kir2.1-regulated resting membrane potential (RMP) is insufficient to alleviate behavioral deficits. Second, CA1 hyperexcitability coupled with E/I imbalance appears to be the primary driver of behavioral abnormalities in these mutant mice. Third, neuronal VPS35/retromer and LEV are critical for sustaining E/I balance; both factors prevent CA1 hyperexcitability and mitigate behavioral deficits, likely via preserving GABAergic synapses. Collectively, these findings demonstrate that impaired GABAergic synapse induces E/I imbalance, which mediates cognitive dysfunction in *Vps35* mutant mice. LEV could restore GABAergic function and network homeostasis following *Vps35* deficiency in excitatory neurons. This study further validates the involvement of retromer dysfunction in AD and ADRD pathogenesis and highlights retromer restoration and LEV treatment as promising therapeutic strategies for these diseases.

## Materials and methods

### Animals

Vps35-floxed (*Vps35*^*f/f*^) mice were generated, maintained, and genotyped as described previously [38, 54, 55]. The CaMKIIα-Cre mice were purchased form the Jackson Laboratory (Strain#: 005359, Donated by Dr. Susumu Tonegawa—Massachusetts Institute of Technology). All these mouse lines were maintained in C57BL/6 strain background for > 6 generations and confirmed by genotyping analysis with PCR. Mice were maintained on a 12 h light–dark cycle with ad libitum access to water and standard food. The investigators were blind to the genotype, treatment, and experimental groups of the animals. All of the experiments with animals were performed with the approval of the Institutional Animal Care and Use Committee of Case Western Reserve University.

### Generation of viruses

Below chemicals were purchased: polyethylenimine (Sigma Aldrich, St. Louis, MO, 765090) and protease inhibitor cocktail (Roche, Basel, Switzerland, 05892970001). The following plasmids were used for AAV virus packaging with AAV-PHP.eB capsid (Addgene, Watertown, MA, #103005) according to a method published in *Nature Protocols* [56]: pENN.AAV.hSyn.HI.eGFP-Cre.WPRE.SV40 (Addgene, #105540), and the pAAV-hSyn-DIO-myc-Kir2.1-T2A-mCherry vector (AAV-hSyn-DIO-Kir_2.1_) engineered from the AAV-DIO-mCherry (Addgene, #50459) construct. The Kir_2.1_ (Addgene, #60661) stop codon was removed and inserted into the backbone by in-Fusion® HD Cloning system (TaKaRa, Kusatsu, Shiga, Japan, 639650).

### Tissue processing and immunofluorescence staining

Mice with indicated genotypes were anesthetized with a mixture of ketamine (100 mg/kg) and xylazine (10 mg/kg) via intraperitoneal (i.p.) injection, and perfused transcardially with 4% paraformaldehyde (PFA; pH 7.4). The brains were dissociated and post-fixed overnight, then mounted in agarose prior to vibratome (Leica, Wetzlar, Germany, vt1000s) sectioning into 40 µm sections. The free-floating sections were collected in PBS, then blocked and permeabilized with blocking solution containing 3% donkey serum and 0.5% Tween-20 in PBS for 1 h at room temperature and incubated overnight with primary antibodies in blocking solution at 4 ℃: guinea pig polyclonal anti-S100β (SYSY, Göttingen, Germany, 287004); mouse monoclonal anti-NeuN (Millipore Sigma, Burlington, MA, MAB377); rabbit monoclonal anti-GFAP (Cell Signaling Technology, Danvers, MA, 12389S); mouse monoclonal anti-Doublecortin/DCX (Santa Cruz Biotechnology, Dallas, TX, sc-271390); rabbit polyclonal anti-Kir2.1/KCNJ2 (Alomone Labs, Jerusalem, Israel, APC-026); rat monoclonal anti-mCherry (Invitrogen, Carlsbad, CA, M11217); mouse monoclonal anti-Myc (Abcam, Cambridge, UK, ab32); mouse monoclonal anti-Gephyrin (SYSY, 147011); chicken monoclonal anti-Vgat (SYSY, 131009); mouse monoclonal anti-PSD95 (Covance, Princeton, NJ, MMS-5182); guinea pig polyclonal anti-Vglut1 (SYSY, 135304); and rabbit monoclonal anti-SV2A (Cell Signaling Technology, 66724S). The brain sections were then rinsed with 3× PBST buffer. Afterward, samples were incubated at room temperature for 2 h with appropriate Alexa Fluor-conjugated secondary antibodies (1:300; Thermo Fisher Scientific, Waltham, MA), including goat anti-guinea pig IgG (H+L) Alexa Fluor 488 (A-11073), Alexa Fluor 594 (A-11076), and Alexa Fluor 647 (A-21450); goat anti-mouse IgG (H+L) Alexa Fluor 488 (A-11001), Alexa Fluor 594 (A-11005), and Alexa Fluor 647 (A-21235); goat anti-rabbit IgG (H+L) Alexa Fluor 488 (A-11008), Alexa Fluor 594 (A-11012), and Alexa Fluor 647 (A-21244); and goat anti-rat IgG (H+L) Alexa Fluor 488 (A-11006), Alexa Fluor 594 (A-11007), and Alexa Fluor 647 (A-21247). Brain sections were washed and incubated with DAPI (Thermo Fisher Scientific, Waltham, MA, D1306) to reveal cell nuclei. After washing with PBS, mouse brain sections were mounted for confocal imaging with the Zeiss LSM 800 system (Carl Zeiss, Oberkochen, Germany). Images were taken with a BZX fluorescence microscope (Keyence, Osaka, Japan, BZ-X1000), as described previously [57].

### RNA-scope-based in situ hybridization

RNA-Scope reagent (RNAscope Multiplex Fluorescent Reagent Kit v2 Assay, 323100), the mouse VPS35 probe, as well as the hybrid oven were purchased from Advanced Cell Diagnostics (ACD, Newark, CA). All procedures were performed according to the manufacturer's protocols as described previously [57]. For RNA-scope data analysis, three brain samples from *Vps35*^*CaMKIIa*^ and littermate control mice were examined, and each brain sample was imaged up to 10 random fields. Images were captured at 40× magnification using a Zeiss confocal microscope, after randomization and decoding of all the images.

### Adapted magnified analysis of the proteome (aMAP)

The MAP procedure was adapted as described previously [58], based on the protocol by Ku et al. [59]. Brain tissues were thoroughly washed and embedded in a hybrid polymer using a MAP solution consisting of 30% acrylamide, 10% sodium acrylate, and 0.1% bis-acrylamide in PBS. Additionally, 10% N,N′-methylenebisacrylamide (VA-50) stock solution was freshly prepared, and 0.03% was added to the final mixture. Following polymerization, the tissues underwent gelation, denaturation, clearing, and expansion, after which immunostaining was performed for MAP imaging. All the clarified or expanded brain tissues were washed with three times of PBST (0.1% Tween-20 in 1× PBS), and immunostaining was carried out for 3 days using a primary antibody. After washing three times with PBST solution for 2 h, gels were incubated with a secondary antibody for 3 days. The labeled gels were washed three times with PBST solution for 2 h and gel expansion was achieved by storing the gel in 0.001× PBS for 1 day in the dark. The expanded brain tissues were mounted onto 0.01 mg/mL PLL-coated high-precision glass coverslips.

### Western blotting

Tissues were dissected and homogenized with RIPA lysis buffer containing a complete protease inhibitor cocktail (Roche, 05892970001). Protein was collected by centrifugation at 13,000× *g* for 5 min. The protein concentration was measured by the BCA protein kit (Thermo Fisher Scientific, Waltham, MA, A55864), and the supernatants were mixed with loading buffer. For western blot analysis, 9% or 11% SDS-PAGE gels were used to separate proteins, and then the proteins were transferred onto nitrocellulose membranes. Afterward, the membranes were incubated with the following primary antibodies: mouse monoclonal anti-Gephyrin (SYSY, 147011); guinea pig polyclonal anti-Vgat (SYSY, 131004); mouse monoclonal anti-β-actin (Sigma-Aldrich, St. Louis, MO, A1978); rabbit polyclonal anti-SV2A (Cell Signaling Technology, 66724S); rabbit polyclonal anti-GABAaR β2 (SYSY, 224803); mouse monoclonal anti-GABAaR β3 (SYSY, 224411); rabbit polyclonal anti-GABAaR γ2 (SYSY, 224003); goat polyclonal anti-VPS35 (Abcam, ab10099); rabbit polyclonal anti-VPS26A (Abcam, ab23892); mouse monoclonal anti-VPS29 (Abcam, ab236796); rabbit polyclonal anti-SNX27 (Abcam, ab241128); rabbit polyclonal anti-Kir2.1 (Alomone Labs, APC-026); mouse monoclonal anti-Hsp30 (Abcam, ab278468); rabbit monoclonal anti-HDAC2 (Cell Signaling Technology, 57156); mouse monoclonal anti-c-Myc (Abcam, ab32); and mouse monoclonal anti-PSD95 (Covance, MMS-5182). This was followed by incubation with species-specific horseradish-peroxidase-conjugated secondary antibodies: rabbit anti-mouse IgG (H+L), HRP (Thermo Fisher Scientific, Waltham, MA, 31452); goat anti-rabbit IgG (H+L), HRP (Thermo Fisher Scientific, Waltham, MA, A16096); and rabbit anti-guinea pig IgG (H+L), HRP (Thermo Fisher Scientific, Waltham, MA, 61-4620). The target proteins were visualized using the Pierce™ ECL Western Blotting Substrate (Thermo Fisher Scientific, Rockford, IL, 32106). Brain tissues from the hippocampus, cortex, and pons of subregional control and *Vps35*^*CaMKIIa*^ mice were fractionated by detergent solubility using a subcellular protein fractionation kit for tissues (Thermo Fisher Scientific, Waltham, MA, 78890). These fractions were then chloroform–methanol precipitated and separated by SDS-PAGE analysis. All data were normalized by β-actin and analyzed using ImageJ software as described previously [57].

### Behavioral tests

All behavioral experiments were conducted with experimenters blinded to genotype and treatment.

Open field test (OFT) was performed as previously described [60]. In brief, mice were individually placed in the center of a square open-field arena (50 × 50 × 20 cm^3^) and allowed to freely explore for 10 min. Locomotor activity was recorded using an overhead video camera. The total distance traveled, the time spent in the center zone, and the number of center entries were quantified using the EthoVision XT software (Noldus Information Technology, Wageningen, The Netherlands).

Elevated plus maze test (EPMT) was conducted as described previously [61]. The apparatus consisted of two open arms and two closed arms with opaque walls (40 cm high), arranged in a plus configuration and elevated above the floor. Mice were placed on the central platform facing an open arm and allowed to explore the maze for 5 min. Behavior was recorded under the same lighting conditions and camera setup as used for the OFT. Time spent in the open arms, closed arms, and center area, as well as total distance travelled, were analyzed using EthoVision XT software.

Novel object recognition (NOR) was performed using a modified protocol based on previously published studies [55, 62]. Mice were habituated to a black Plexiglas open-field arena (50 × 50 × 20 cm^3^) for 10 min per day over two consecutive days under dim ambient lighting. Behavioral activity was recorded using an overhead video camera. The test consisted of an acquisition phase followed by a testing phase. During acquisition, two identical objects were placed in diagonally opposite corners of the arena, 8–9 cm from the walls. Mice were placed in the center of the arena and allowed to explore the objects for 10 min before being returned to their home cages. The testing phase was conducted 4 h after acquisition, during which one familiar object was replaced with a novel object of identical height and volume but different shape and appearance. Mice were allowed to explore the objects for 5 min. Object exploration was defined as nose sniffing or head orientation within 1 cm of the object and was scored by experimenters blinded to genotype and treatment. The discrimination index was calculated as (*T*_new_ − *T*_old_)/(*T*_new_ + *T*_old_), where ${T}_{new}$ represents the time spent exploring the novel object or location, and ${T}_{old}$ represents the time spent exploring the familiar object or location. Each genotype was pretested to exclude intrinsic object preference.

Morris water maze (MWM) was performed according to the original method described by Morris [63]. Mice were trained in a circular pool (52 cm diameter) filled with water in a room containing prominent extra-maze visual cues. Each mouse received four training trials per day for 5 consecutive days with an intertrial interval of 15 min. The maximum duration of each trial was 60 s. Mice that failed to locate the hidden platform within this time were gently guided to the platform and allowed to remain there briefly. A probe trial was conducted 24 h after the final training session, during which the platform was removed. The percentage of time spent in the target quadrant that previously contained the platform was recorded during a 60-s trial. Escape latency and swimming speed were analyzed using EthoVision XT software (Noldus Information Technology). Body weight, visual ability, and swimming speed were monitored to control potential motor or sensory confounding effects.

Tail suspension test (TST) was performed using a custom-made tail-suspension apparatus (55 × 60 cm^2^) divided into four compartments (15 cm each). Within each compartment, a plastic bar was suspended from the top. Mice were suspended by taping the tail to the bar and recorded for 6 min. Immobility was defined as the absence of escape-oriented movements. Percentage immobility was calculated as the total duration of immobility divided by the total test duration.

Forced swim test (FST) was performed as previously described [55]. Mice were individually placed in a transparent plastic cylinder (12 cm diameter, 25 cm height) filled with room-temperature water (23–24 °C) to a depth sufficient to prevent contact with the bottom using the tail or hind limbs. Cylinders were visually isolated to prevent animals from observing neighboring mice. Mice were allowed to swim for 6 min under standard illumination (100–200 lx). After testing, mice were removed from the water, gently dried, and returned to a warmed cage. Behavior was recorded from the side. Immobility during the final 4 min of the test was scored offline by an observer blinded to genotype and treatment. Immobility was defined as the absence of active escape-directed behaviors, with mice making only minimal movements necessary to maintain balance and keep the head above water.

### Drug treatments

LEV (Millipore Sigma, L8668), LAMO (Tocris Bioscience, Bristol, UK, 1611) and VPA (Tocris Bioscience, 2815) were dissolved in water or DMSO, and then diluted with sterile saline solution (0.9% NaCl) to make stock solution. Control and *Vps35*^*CaMKIIa*^ mice were randomly assigned to vehicle or treatment groups and received daily intraperitoneal injections as follows: LEV (75 mg/kg for 15 consecutive days), LAMO (20 mg/kg for 21 consecutive days), or VPA (30 mg/kg for 30 consecutive days). Following the final injection, mice were subjected to behavioral testing, electrophysiological recordings, or were anesthetized and transcardially perfused with cold PBS. Brain tissues were collected for immunohistochemical and biochemical analyses.

### EEG recording and analysis

EEG recordings were performed as described previously [64]. To record hippocampal local field potentials (LFPs), EEG recording electrodes were placed at the following coordinates relative to bregma: AP, −1.85 mm; ML, ±1.25 mm; and DV, −1.95 mm, and were fixed to the skull with screws. A screw placed over the cerebellum served as the ground electrode. The electrodes and screws were secured to the skull using dental cement. After a 7-day recovery period, each mouse was recorded in a home cage with ad libitum access to food and water. EEG signals were amplified using a differential bio-amplifier (AD Instruments, Colorado Springs, CO, FE136) and digitized with a PowerLab system (AD Instruments). Signals were sampled at 1 kHz with a 0.3 Hz high-pass filter and a 100 Hz low-pass filter. As reported previously, the frequency of epileptic spikes was counted to evaluate abnormal brain activity. EEG spikes were defined as paroxysmal sharp waves with a duration of 20–150 ms and an amplitude > 2 times that of the background EEG activity.

### Electrophysiology recordings in acute hippocampal slices

Acute hippocampal slices were prepared as previously described [61, 65] with minor modifications. Mice were deeply anesthetized with isoflurane and decapitated, and brains were rapidly removed and immersed in ice-cold, oxygenated (95% O₂/5% CO₂) cutting solution containing (in mM): 120 choline chloride, 2.5 KCl, 7 MgCl₂, 0.5 CaCl₂, 1.25 NaH₂PO₄, 26 NaHCO₃, and 25 glucose. Transverse hippocampal slices (320 μm) were cut using a VT1200S vibratome (Leica Microsystems). Slices recovered for 30 min at 32 °C in oxygenated artificial cerebrospinal fluid (ACSF) containing (in mM): 124 NaCl, 2.5 KCl, 2 MgSO₄, 2.5 CaCl₂, 1.25 NaH₂PO₄, 26 NaHCO₃, and 10 glucose, and were subsequently maintained at room temperature (25 ± 1 °C) for at least 1 h before recording.

For recordings, slices were transferred to a submerged chamber and continuously superfused with oxygenated ACSF (2 mL/min) at 32–34 °C. CA1 pyramidal neurons were visually identified using infrared differential interference contrast optics on an upright microscope (Olympus, Waltham, MA, BX51WI) equipped with a 40× water-immersion objective and a CCD camera (Hamamatsu, Bridgewater, NJ). Patch pipettes (3–5 MΩ) were pulled using a horizontal puller (Sutter Instruments, Novato, CA, P-1000). Signals were acquired with a MultiClamp 700B amplifier and digitized using a Digidata 1550A (Molecular Devices, San Jose, CA), low-pass filtered at 2.2 kHz, and sampled at 10 kHz.

Whole-cell current-clamp recordings were performed to assess intrinsic excitability and resting membrane potential (RMP). Patch pipettes were filled with a potassium-based internal solution containing (in mM): 125 K-gluconate, 5 KCl, 10 HEPES, 0.2 EGTA, 1 MgCl₂, 4 Mg-ATP, 0.3 Na-GTP, and 10 phosphocreatine (pH 7.35; 290 mOsm). After achieving a high-resistance seal (≥ 1 GΩ), the membrane was ruptured to obtain whole-cell configuration. Recordings were conducted at zero holding current (*I* = 0 pA), with bridge balance and pipette capacitance compensated. Cells were excluded if series resistance exceeded 25 MΩ or if input resistance varied by more than 20% during recording. RMP was measured immediately after break-in and monitored for 1–2 min until stabilization. For each neuron, RMP was calculated as the mean membrane potential over a stable 20–30 s period. Cells exhibiting membrane potential fluctuations >3 mV were excluded. Intrinsic excitability was assessed by injecting depolarizing current steps of increasing amplitude (1 s duration, 20 s inter-sweep interval), and the number of evoked action potentials was quantified.

Spontaneous excitatory postsynaptic currents (sEPSCs) were recorded from CA1 pyramidal neurons in whole-cell voltage-clamp mode at − 70 mV in the presence of bicuculline (20 μM). Miniature EPSCs were recorded following addition of tetrodotoxin (TTX, 1 μM).

Miniature inhibitory postsynaptic currents (mIPSCs) were recorded in the presence of TTX (1 μM), CNQX (20 μM), and DL-AP5 (50 μM) to block action potentials and glutamatergic transmission. For spontaneous IPSC (sIPSC) recordings, TTX was omitted.

In a subset of experiments, sEPSCs and sIPSCs were sequentially recorded from the same neuron, with sEPSCs recorded at −70 mV and sIPSCs at 0 mV. Event frequency was quantified during stable recording periods, and the E/I ratio was calculated for each cell as the ratio of sEPSC frequency to sIPSC frequency.

Evoked synaptic responses were elicited using a bipolar stimulating electrode placed in the Schaffer collateral (SC)–CA1 pathway. Whole-cell voltage-clamp recordings were performed at holding potentials of − 70 mV and 0 mV to isolate EPSCs and IPSCs, respectively. Synaptic current identities were pharmacologically verified using bicuculline (20 μM) and CNQX/DL-AP5 (20 μM/100 μM). EPSCs and IPSCs were quantified by peak amplitude or charge transfer.

Paired-pulse ratio (PPR) recordings were performed by stimulating the SC pathway with paired pulses delivered at interstimulus intervals of 25, 50, 100, and 200 ms. EPSCs or IPSCs were recorded at − 70 mV in the presence of bicuculline or CNQX/DL-AP5, respectively. PPR was calculated as P2/P1, where P1 and P2 denote the amplitudes of the first and the second synaptic responses.

Inwardly rectifying K^+^ (Kir) currents were recorded in the whole-cell voltage-clamp mode using 300-ms voltage steps from − 140 to + 10 mV in 10 mV increments, preceded by a 15 ms prepulse at − 20 mV. The external solution contained (in mM): 134 NaCl, 6 KCl, 1 MgCl₂, 2 CaCl₂, 10 HEPES, and 10 glucose (pH 7.4). To isolate Kir currents, CdCl₂ (0.4 mM), TTX (20 μM), 4-AP (1 mM), and glyburide (10 μM) were included. The internal solution contained (in mM): 110 potassium aspartate, 30 KCl, 10 NaCl, 10 HEPES, 5 EGTA, and 1 MgCl₂ (pH 7.2). Kir currents were confirmed by sensitivity to Ba^2+^ (100 μM).

Voltage-gated Na^+^ currents were recorded using an internal solution containing (in mM): 120 CsF, 10 NaCl, 10 HEPES, 10 EGTA, 4 Mg-ATP, and 0.3 Na-GTP (pH 7.3; 295–300 mOsm). The external solution contained TEA (20 mM), 4-AP (5 mM), and CdCl₂ (100 μM). Cells were held at −80 mV, and Na^+^ currents were evoked by 20 ms depolarizing steps from − 80 to + 50 mV. Peak inward currents were measured within the first 1–3 ms. Linear leak and capacitive currents were subtracted using a P/4 protocol, and Na^+^ current identity was confirmed by TTX (1 μM). Liquid junction potentials (~ −10 mV) were corrected offline.

Field excitatory postsynaptic potentials (fEPSPs) were recorded in the stratum radiatum of the CA1 region using ACSF-filled glass microelectrodes (2–5 MΩ), with synaptic responses evoked by electrical stimulation of the SC pathway via a bipolar stimulating electrode. Basal synaptic transmission was assessed by generating input–output (I–O) relationships. Monophasic constant-current pulses (100 μs duration) of increasing intensity were delivered at a low frequency (0.033 Hz), and fEPSPs were recorded at each stimulation level. Synaptic strength was quantified as the slope of the initial rising phase of the fEPSP (10%–60%), and I–O curves were constructed by plotting fEPSP slope as a function of stimulation intensity. For long-term potentiation (LTP) experiments, a stable baseline of fEPSPs was recorded for at least 20 min at the baseline stimulation intensity. LTP was induced using a high-frequency stimulation (HFS) protocol consisting of two trains of 100 Hz stimulation (1 s duration each), delivered with a 30 s inter-train interval. Following HFS, fEPSPs were monitored for 60 min at the baseline stimulation frequency. Changes in synaptic efficacy were expressed as the percentage change in fEPSP slope relative to the pre-induction baseline. Representative fEPSP traces were averaged from responses collected during the baseline period and during the late phase of LTP, as indicated in Fig. 3f and g [66].

Tonic GABA_A_ receptor-mediated currents were recorded in whole-cell voltage-clamp mode at − 70 mV and 33 °C. Recordings were performed in the presence of DL-APV (20 μM), DNQX (20 μM), and CGP55845 (1 μM) to block ionotropic glutamate receptors and GABA_B_ receptors. To activate extrasynaptic GABA_A_ receptors, GABA (5 μM) was included in the ACSF. Tonic currents were quantified as the change in holding current following bath application of picrotoxin.

### In vivo electrophysiological recordings of hippocampal place cells

Mice were anesthetized with ketamine/xylazine and implanted with four tetrodes targeting the CA1 region of the right hippocampus (AP − 2.0 mm, ± ML 1.5 mm, DV − 1.3 mm) using standard stereotaxic procedures. Tetrodes were constructed from platinum-iridium wire and mounted on a movable microdrive. Stainless steel skull screws served as ground and reference electrodes. Following at least 1 week of recovery, neural activity was recorded while mice were freely exploring an open-field arena (80 × 80 × 20 cm^3^).

Neural signals were acquired using the OmniPlex system (Plexon, Dallas, TX). Local field potentials were filtered at 0.5–1000 Hz and digitized at 1 kHz, and spike signals were filtered at 0.6–6 kHz and digitized at 40 kHz. Animal position was tracked using ANY-maze software and synchronized with electrophysiological recordings. Spike sorting was performed offline using MClust with a semi-automated superparamagnetic clustering algorithm. Only well-isolated single units with a spike width > 300 μs and a peak firing rate > 1 Hz were included for analysis.

Place field analyses were performed using NeuroExplorer. Position data were binned into 1.5 × 1.5 cm grids, and firing-rate maps were generated by normalizing spike counts to occupancy time and smoothed using a 5 × 5 boxcar filter. Place field size and spatial firing properties were quantified from the smoothed rate maps.

### Statistical analysis

Statistical analyses were performed using GraphPad Prism (version 10.1). Data are presented as mean ± SEM unless otherwise stated. For comparisons between two independent groups, two-tailed unpaired Student’s *t* tests were used. For paired comparisons, two-tailed paired Student’s *t* tests were applied when appropriate. For comparisons involving more than two groups, one-way or two-way ANOVA was used, followed by Sidak’s multiple comparisons test. Nonparametric datasets were analyzed using the Kruskal–Wallis test followed by Dunn’s multiple comparisons test. Repeated-measures datasets, including input–output relationships, frequency-current curves, synaptic plasticity time courses, paired-pulse ratio curves, and behavioral training data, were analyzed using mixed-effects models with restricted maximum likelihood (REML). Post-hoc comparisons were performed using Sidak’s correction, as specified in the figure legends. Cumulative probability distributions (e.g., inter-event interval and amplitude distributions) were compared using the Kolmogorov–Smirnov test. For place cell analyses, statistical tests were applied as indicated in the corresponding figure legends, including unpaired *t* tests or nonparametric tests when appropriate. Statistical significance was set at *P* < 0.05.

## Results

### Progressive cognitive decline in *Vps35*^*CaMKIIa*^ mice

To investigate the functions of neuronal VPS35 in post-developmental ages, we generated *Vps35*^*CaMKIIa*^ mice by crossing Vps35-flox (*Vps35*^*f/f*^) mice with CaMKIIa-Cre mice, using *Vps35*^*f/f*^ littermates as controls. This strategy selectively knocked out (KO) VPS35 in CaMKIIa-Cre positive (Cre^+^) PyNs of the adult hippocampus and cortex. As shown in Fig. S1a-b, levels of VPS35 and the associated retromer components VPS26a and VPS29, were markedly reduced in the mutant hippocampus, confirming the loss of retromer complex. The residual VPS35 detected by Western blot likely reflects the expression in non-pyramidal cell types, such as glia and vascular cells, as our RNAscope analysis showed depletion of *Vps35*-mRNAs in NeuN^+^ neurons, but not in NeuN^−^ cells, in *Vps35*^*CaMKIIa*^ CA1 region (Fig. S1c, d). Despite the loss of VPS35 in PyNs, mutant mice exhibited normal lifespan and body weight (Fig. S1e) and showed no major alterations in neuronal or glial cell density and distribution in the hippocampus and cortex during early adulthood (Fig. S1f-k). This contrasts sharply with *Vps35*^*NeuroD6*^ mice, in which VPS35 depletion during PyN development leads to pronounced structural abnormalities [40].

**Fig. 1:**
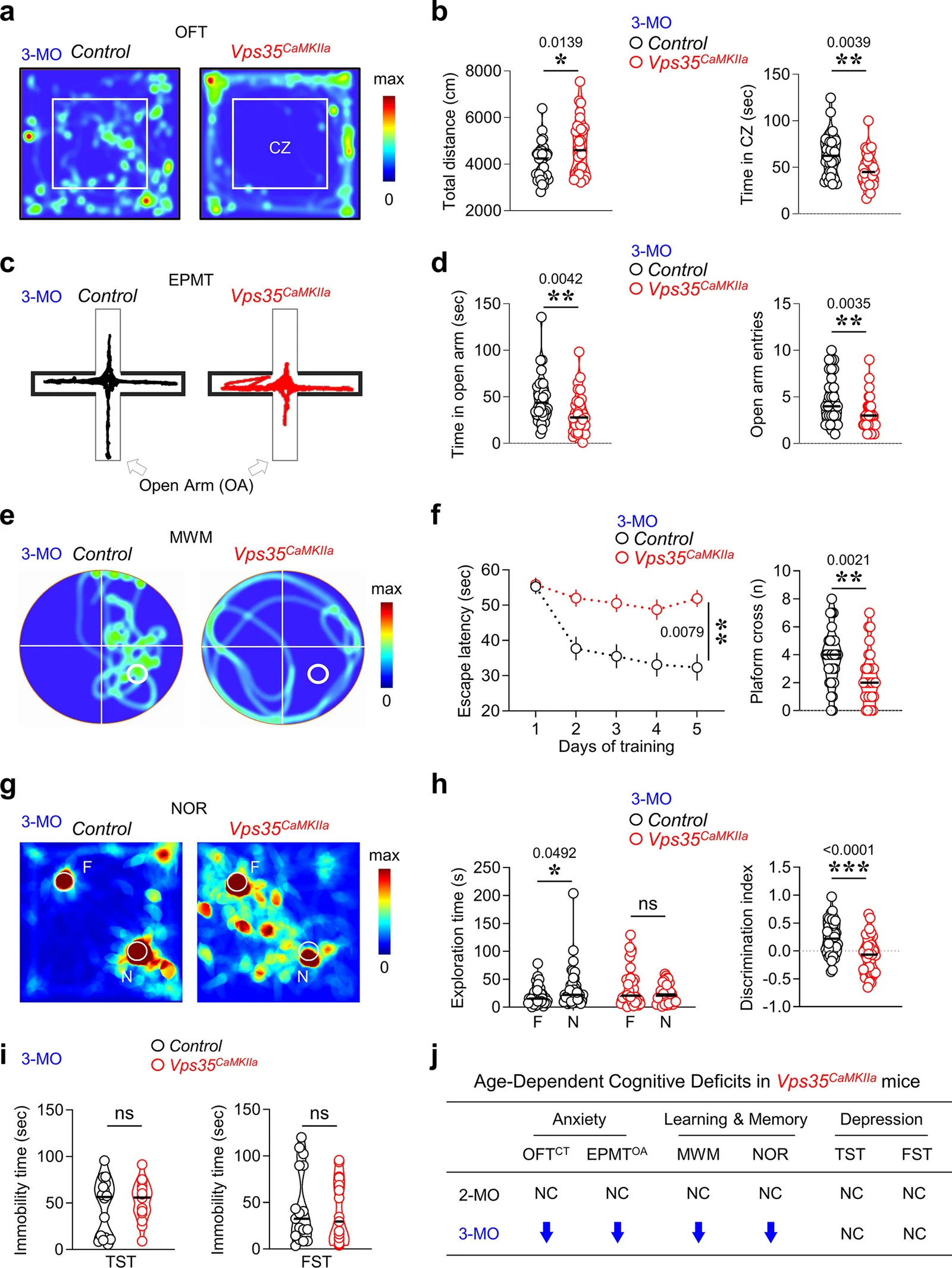
Altered anxiety-related behaviors and learning and memory performance of *Vps35*^*CaMKIIa*^ mice and littermate controls at age of ≥ 3 months (3-MO). **a, b** Representative heat maps of locomotor activity and quantifications of total distance traveled and time spent in the center area in the open field test (OFT). **c, d** Representative movement trajectories, and quantifications of open-arm duration and open-arm entries in the elevated plus maze test (EPMT). **e, f** Representative heat maps from the Morris water maze (MWM) probe trial, and quantifications of escape latency during the training phase (left) and number of platform crossings during the probe trial (right). **g, h** Representative exploration heat maps in the novel object recognition (NOR) test, and quantifications of exploration time for familiar (F) and novel objects (N) and discrimination index. **i** Immobility time during the tail suspension test (TST; left) and forced swim test (FST; right). All quantifications are presented as mean ± SD. Each dot represents one mouse (*n* = 30–40 mice per group). Escape latency during MWM training was analyzed using a mixed-effects model (REML), with training day as a repeated factor and genotype as a between-subject factor, followed by Sidak’s multiple comparisons test. Exploration time during the NOR test was analyzed using two-way ANOVA with object type and genotype as factors, followed by Sidak’s multiple comparisons test. For all other behavioral measures, data normality and variance homogeneity were assessed before statistical testing; unpaired two-tailed Student’s *t*-test, Welch’s t-test, or Mann–Whitney test was used as appropriate. *P < 0.05; **P < 0.01; ***P < 0.001; ns, not significant. **j** Summary of age-dependent behavioral phenotypes in *Vps35*^*CaMKIIa*^ mice

To determine whether *Vps35*^*CaMKIIa*^ mice develop AD/ADRD-relevant functional deficits, we initially performed a series of behavioral tests in control and *Vps35*^*CaMKIIa*^ male mice at 3 month old (MO). The *Vps35*^*CaMKIIa*^ mice exhibited increased locomotor activity, indicated by greater distances traveled in the OFT, but spent less time in the center of the arena, suggestive of anxiety-like behavior (Fig. 1a, b). This anxiety-like phenotype was corroborated by the EPMT results, where the mutant mice made fewer entries into and spent less time in the open arms compared to controls (*Vps35*^*f/f*^) (Fig. 1c, d).

Cognitive testing also revealed significant impairments in *Vps35*^*CaMKIIa*^ mice. In the MWM, mutant mice exhibited deficits in spatial learning and memory (Fig. 1e, f), while in the NOR test, they showed reduced exploration of novel objects (Fig. 1g, h). The reduced exploration of novel objects was not due to the position bias, given that the mice showed no significant preference for any corner during the object preference test or for any object during the training phase with two identical objects (Fig. S2). Additionally, minimal differences in the TST and FST were found between mutant and control mice at 3-MO (Fig. 1i). These results thus indicate impaired learning and memory in the mutant mice.

We then assessed whether these behavior deficits occurred in 3-MO female mutant mice, and found similar behavioral (such as NOR) deficits as those in male mutant mice (Fig. S4). Interestingly, these behavior changes were not evident in 2-MO mutants (Fig. S3). Together, these findings (Fig. 1j) suggest that *Vps35*^*CaMKIIa*^ mice develop progressive behavioral changes in young adult age, including anxiety and impaired learning and memory, mirroring the behavior phenotypes reported in aged AD models (e.g., J20, APP/PS1) [25, 67].

### Earlier-onset hyperexcitability and depolarized RMP in *Vps35*^*CaMKIIa*^ hippocampal CA1 neurons

**Fig. 2:**
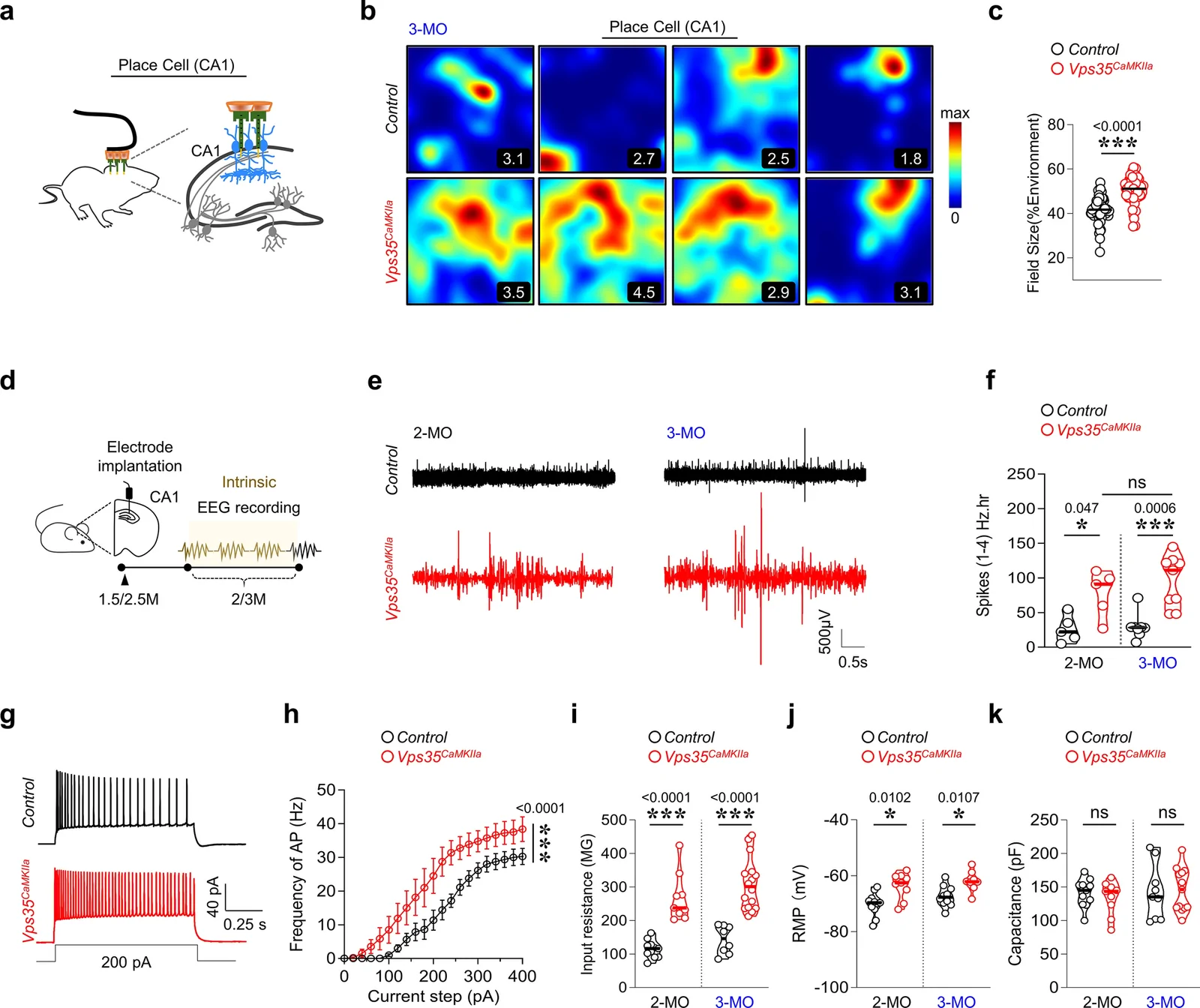
Disrupted hippocampal place cell coding and heightened CA1 pyramidal neuron excitability in *Vps35*^*CaMKIIa*^ mice. **a** Schematic illustration of in vivo extracellular recordings of CA1 hippocampal place cells in freely moving mice. **b** Representative spatial firing rate maps of CA1 place cells recorded from 3-MO control and *Vps35*^*CaMKIIa*^ mice. Dark red indicates higher spike-firing rates, whereas blue indicates lower spike-firing rates. Numbers in the lower right corner indicate the peak firing rate of each place cell. **c** Quantification of place cell field size reveals an increased spatial coverage in *Vps35*^*CaMKIIa*^ mice compared with controls (control, *n* =69 place cells; *Vps35*^*CaMKIIa*^, *n* =57 place cells). **d** Experimental timeline for electrode implantation and electroencephalogram (EEG) recordings. **e** Representative EEG traces recorded from 2-MO and 3-MO littermate control and *Vps35*^*CaMKIIa*^ mice. **f** Quantification of epileptic spike events during 1-h EEG recordings in 2-MO and 3-MO littermate control and *Vps35*^*CaMKIIa*^ mice. **g–k** Representative voltage responses of CA1 pyramidal neurons evoked by 1-s current depolarizing current injections during whole-cell current-clamp recordings in acute hippocampal slices (**g**), and firing frequency plotted against injected current intensity (**h**). Input resistance (**i**), resting membrane potential (**j**), and membrane capacitance (**k**) were measured in CA1 pyramidal neurons from 2-MO and 3-MO littermate control and *Vps35*^*CaMKIIa*^ mice. All quantifications are presented as mean ± SD. Each dot represents one neuron or one mouse, as indicated. Place-cell field size was analyzed using an unpaired two-tailed Student’s *t*-test. Action potential firing frequency was analyzed using a mixed-effects model (REML), with current injection step as a repeated measure and genotype as a between-subject factor, followed by Sidak’s multiple-comparisons test (*n* = 9–12 neurons per genotype, obtained from at least three mice). In vivo EEG spike rates and all other electrophysiological parameters were analyzed using one-way ANOVA with age and genotype as factors, followed by Tukey’s multiple-comparisons test (EEG recordings: 2 months, *n* = 5 mice per genotype; 3 months, *n* = 8 control mice and *n* = 9 *Vps35*^*CaMKIIa*^). *P < 0.05; **P < 0.01; ***P < 0.001; ns, not significant

Given the well-established association between cognitive impairment and hippocampal neuronal excitability dysregulation [68, 69], we investigated whether *Vps35*^*CaMKIIα*^ mice exhibit heightened firing. We initially performed in vivo recordings of hippocampal CA1 neurons, monitoring the activity of individual neurons as mice explored open-field environments (Fig. 2a). Compared to controls, the mutant mice showed enlarged firing fields in the CA1 region when entering specific spatial locations (Fig. 2b, c), consistent with altered place cell activity [70, 71]. This heightened firing pattern was detectable in some of the 2-MO mutant mice and became more pronounced at 3-MO (Fig. 2b, c), suggesting a progressive increase in CA1 place cell firing that may contribute to emerging spatial memory deficits.

To further assess network-level excitability, we conducted intracranial EEG recordings, given the established link of hippocampal hyperexcitability with seizure susceptibility [20, 72]. Electrodes were implanted in the dorsal hippocampus of both control and *Vps35*^*CaMKIIα*^ mice at 1.5 or 2.5 months of age, enabling continuous monitoring at 2 or 3 months, respectively (Fig. 2d). Mutant mice exhibited significantly elevated epileptiform spike activity at both ages, with a more pronounced increase at 3 months compared to controls (Fig. 2e, f), providing additional in vivo evidence of hippocampal hyperexcitability.

We then examined the intrinsic electrophysiological properties of CA1 neurons in acute brain slices. At 2-MO, *Vps35*^*CaMKIIα*^ CA1 neurons exhibited increased firing rates, elevated input resistance, and a more depolarized RMP, while membrane capacitance remained unchanged (Fig. 2g–k). These changes were already evident as early as 2-MO and persisted at later ages (Fig. 2i, j), indicating that neuronal hyperexcitability is an early, progressive, and sustained phenotype in *Vps35*^*CaMKIIα*^ mice.

### Reduced potassium channel and currents contributing to the CA1 hyperexcitability with limited impact on cognition decline in *Vps35*^*CaMKIIa*^ mice

To understand how VPS35 loss in PyNs impairs RMP and induces hyperexcitability, we measured voltage-dependent Na^+^ and K^+^ currents in CA1 PyNs, as both currents are crucial for maintaining RMP and regulating neuronal excitability [73, 74]. Using whole-cell voltage-clamp recordings, we found that the Na^+^ currents in mutant CA1 PyNs were comparable to those in control neurons (Fig. S5a), ruling out Na^+^ current hyper-function as a contributor to the hyperexcitability phenotype. However, a significant reduction in inward rectifier K^+^ current was detected in 2-MO mutants (Fig. S5b), suggesting that the impaired K^+^ current may contribute to the observed RMP and excitability abnormalities.

We thus assessed the expression levels of key transmembrane proteins that may regulate potassium current, such as Kir_2.1_ [75, 76], in the hippocampus of *Vps35*^*CaMKIIα*^ mice (Fig. S5c). Western blot analyses of purified cytoplasmic, membrane, and nuclear fractions from mouse brain homogenates (1-MO) revealed that Kir_2.1_ levels were decreased in the membrane fraction but increased in the cytoplasmic fraction of the mutant hippocampus and cortex, with no significant changes observed in the mutant pons, where VPS35 was not depleted (Fig. S5c, d). Notably, in addition to VPS35, VPS26a levels were reduced, but VPS29 levels remained unchanged, in the 1-MO mutant hippocampus (Fig. S5c, e), unlike that in 3-MO mutant hippocampus (Fig. S1a, b), suggesting a progressive disruption in the retromer complex. Further validation via co-immunostaining analysis showed reduced Kir_2.1_ in CA1 neurons, including both the soma and the apical dendrites (Fig. S5f, g). Collectively, these results indicate that VPS35 loss impairs Kir_2.1_ distribution and activity in CA1 PyNs, positioning Kir_2.1_ as a potential cargo of the VPS35/retromer complex in these neurons.

Given that the *Vps35*^*CaMKIIα*^ hippocampus showed loss of VPS35 and Kir_2.1_ expression as early as 1-MO (Fig. S5c–g), it is possible that the behavior deficits detected in 3-MO mutant mice were due to an impairment in neuronal synapse maturation and/or maintenance. To investigate the latter possibility, we injected AAV-hSyn-Cre or a control virus (AAV-hSyn-GFP) specifically into the hippocampus of *Vps35*^*f/f*^ mice at 2-MO to delete VPS35 expression in young adult age (Adult-KO) and thus avoiding the neonatal developmental defect (Fig. S6a). Electrophysiological recordings and behavior tests at 3-MO showed that the adult-Vps35-KO by AAV-hSyn-Cre also developed heightened firing activity with a more depolarized RMP and increased E/I ratio in the mutant CA1 neurons, as well as impaired novel object recognition (Fig. S6). These findings support that the loss of VPS35 in young adult CA1 neurons is sufficient to induce similar deficits as those in *Vps35*^*CaMKIIα*^ mice. This is in line with the critical roles of young adult CA1 neuronal VPS35 in maintaining the CA1 neuronal RMP and network E/I balance to prevent hyperexcitability and promote E/I balance and cognition.

To investigate whether the earlier Kir_2.1_ loss contributes to the deficits observed in *Vps35*^*CaMKIIα*^ mice, we generated an AAV-hSyn-DIO-Myc-Kir_2.1_-T2A-mCherry virus (designated AAV-DIO-Kir_2.1_), which enables Cre-dependent expression of Myc-tagged Kir_2.1_ in neurons (Fig. S7a–c). We then injected AAV-DIO-Kir_2.1_ or a control virus (AAV-hSyn-DIO-mCherry) into the hippocampus of *Vps35*^*CaMKIIα*^ mice and assessed their effects on CA1 neuron activity. Electrophysiological recordings showed that Kir_2.1_ expression significantly suppressed the heightened firing activity in *Vps35*^*CaMKIIα*^ CA1 PyNs, leading to reduced action potential frequency and a more hyperpolarized RMP (Fig. S7d–f). Additionally, Kir_2.1_ expression inhibited epileptiform discharges (Fig. S7g, h), but had little effect on the E/I imbalance in the mutant hippocampus (Fig. S7i, j). These findings suggest that the loss of Kir_2.1_ may contribute to the hyperexcitability, but not the E/I imbalance, observed in *Vps35*^*CaMKIIα*^ CA1 neurons.

We next determined whether suppressing hippocampal neuronal hyperexcitability by restoring Kir_2.1_ could mitigate the cognitive decline in *Vps35*^*CaMKIIa*^ mice. We injected AAV-DIO-Kir_2.1_ or control virus into both sides of the hippocampus of the mutant mice at 2-MO, and accessed their cognitive function at 3-MO using the MWM, NOR, and OFT. As shown in Fig. S8a–h, Kir_2.1_ expression had minimal impact on learning and memory as assessed by the NOR and MWM tests, compared to control virus-injected mutant mice. In the OFT, Kir_2.1_ expression reduced the increased total distance traveled by the mutant mice, but had little impact on the time spent in the central zone (Fig. S8g, h).

Together, these results (Fig. S8i) suggest that while Kir_2.1_ expression in the mutant hippocampus reduces heightened excitability, RMP, and locomotor activity, it does not significantly alleviate the E/I imbalance, cognitive decline or anxiety behaviors in the mutant mice.

### Linking E/I imbalance to impairments in LTP, anxiety, and cognition

**Fig. 3:**
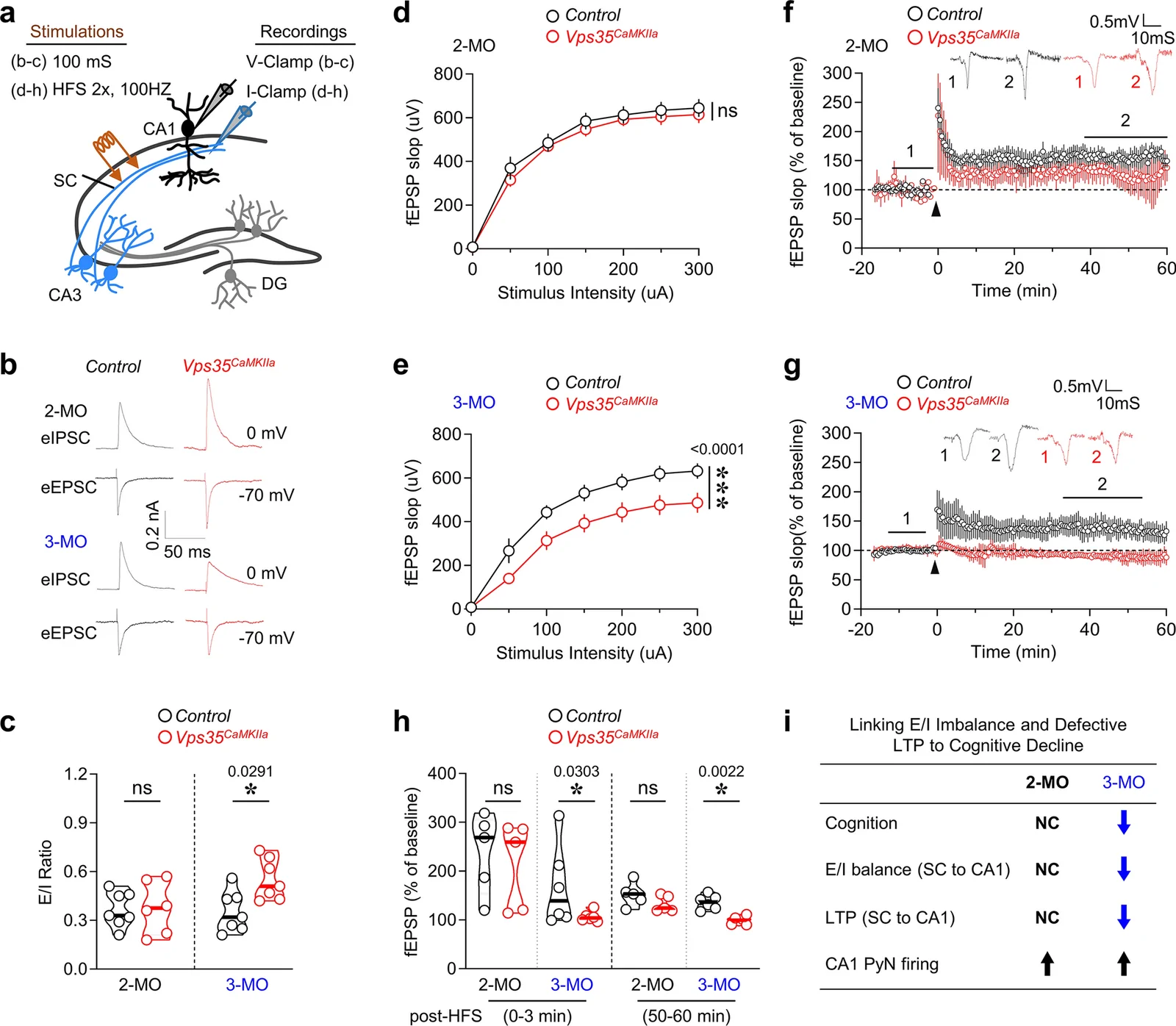
Altered synaptic plasticity and excitation/inhibition balance in the mutant CA1 hippocampus. **a** Schematic illustration of evoked excitatory postsynaptic current (eEPSC), evoked inhibitory postsynaptic current (eIPSC), and field long-term potentiation (LTP) recordings in the hippocampal CA1 region. **b-c** Representative traces of eEPSCs and eIPSCs recorded from CA1 pyramidal neurons from 2-MO and 3-MO littermate control and *Vps35*^*CaMKIIa*^ mice (**b**), and quantification of the evoked EPSC/IPSC ratio (**c**). **d-e** Input–output relationships generated by stimulating Schaffer collaterals at 2-MO (**d**), and 3-MO (**e**). **f, g** Time course of fEPSP slopes recorded before and after high-frequency stimulation (HFS) at Schaffer collateral-CA1 synapses in control and *Vps35*^*CaMKIIa*^ mice at 2-MO (**f**) and 3-MO (**g**). LTP was induced by two trains of 100-Hz stimulation. Insets show representative fEPSP traces recorded before and 50 min after high-frequency stimulation (HFS, black arrow). **h** Quantification of fEPSP slope normalized to baseline during the early (0–3 min) and late (50–60 min) phases after HFS in slices from 2-MO and 3-MO littermate control and *Vps35*^*CaMKIIa*^ mice. All quantifications are presented as mean ± SD. Each dot represents one neuron or one slice, as indicated. Input–output relationships and LTP time courses were analyzed using mixed-effects models (REML) followed by Sidak’s multiple comparisons test. Evoked E/I ratios and late-phase LTP magnitudes (50–60 min post-HFS) were analyzed using two-way ANOVA followed by Sidak’s multiple comparisons test. *P < 0.05; **P < 0.01; ***P < 0.001; ns, not significant. **i** Summary of the age-dependent emergence of E/I imbalance and defective hippocampal LTP and their association with cognitive decline in *Vps35*^*CaMKIIa*^ mice. Arrows indicate the direction of change relative to age-matched controls. All conclusions are based on statistically analyzed data presented in panels **a–h** and Fig. 2g–k

We further explored whether the E/I balance, a key mechanism that ensures circuit stability, prevents seizure activity, and supports synaptic plasticity [77, 78], is progressively impaired in *Vps35*^*CaMKIIa*^ mice. Using voltage-clamp recordings, we measured excitatory and inhibitory synaptic currents in the SC–CA1 pathway from hippocampal slices of control and *Vps35*^*CaMKIIα*^ mice (Fig. 3a). At 2-MO, the E/I ratio in mutant CA1 neurons was similar to controls, but by 3-MO, it was significantly increased (Fig. 3b, c), indicating a progressive disruption in E/I balance.

We next evaluated the basal synaptic transmission and LTP in the SC–CA1 pathway of the hippocampus, as both are also fundamental electrophysiological processes underlying learning and memory [79–81] and often impaired in various AD/ADRD animal models [82, 83]. Assessing the basal synaptic function by recording fEPSPs in the CA1 stratum radiatum following stimulation of the SC pathway at varying intensities showed no significant difference in fEPSP slopes between *Vps35*^*CaMKIIα*^ and littermate control mice at 2-MO (Fig. 3d); however, at 3-MO, the *Vps35*^*CaMKIIα*^ mice exhibited a significant reduction (Fig. 3e), supporting the view for an age-dependent synaptic transmission deficit in the mutants.

Further examining LTP at the SC–CA1 pathway of control and *Vps35*^*CaMKIIa*^ mice, which was induced by HFS and recorded by current clamp (Fig. 3a), showed again a slight reduction, but not significantly different, in 2-MO mutant hippocampal slices (Fig. 3f, h); however, at 3-MO, the LTP was significantly impaired in *Vps35*^*CaMKIIa*^ mice (Fig. 3g, h), providing additional evidence of a progressive decline in synaptic plasticity in the mutant SC-CA1 hippocampus.

Taken together (Fig. 3i), these findings indicate an age-dependent disruption in E/I balance, which coincides with the defective LTP and the onset of anxiety-like behaviors and cognitive decline. This temporal pattern differs from the earlier emergence of CA1 neuronal hyperexcitability (Fig. 2), suggesting distinct timelines for excitability and E/I imbalance or impaired synaptic plasticity in *Vps35*^*CaMKIIα*^ mice.

### Restore of the E/I balance and cognitive functions in 3-MO *Vps35*^*CaMKIIa*^ mice by LEV treatments

**Fig. 4:**
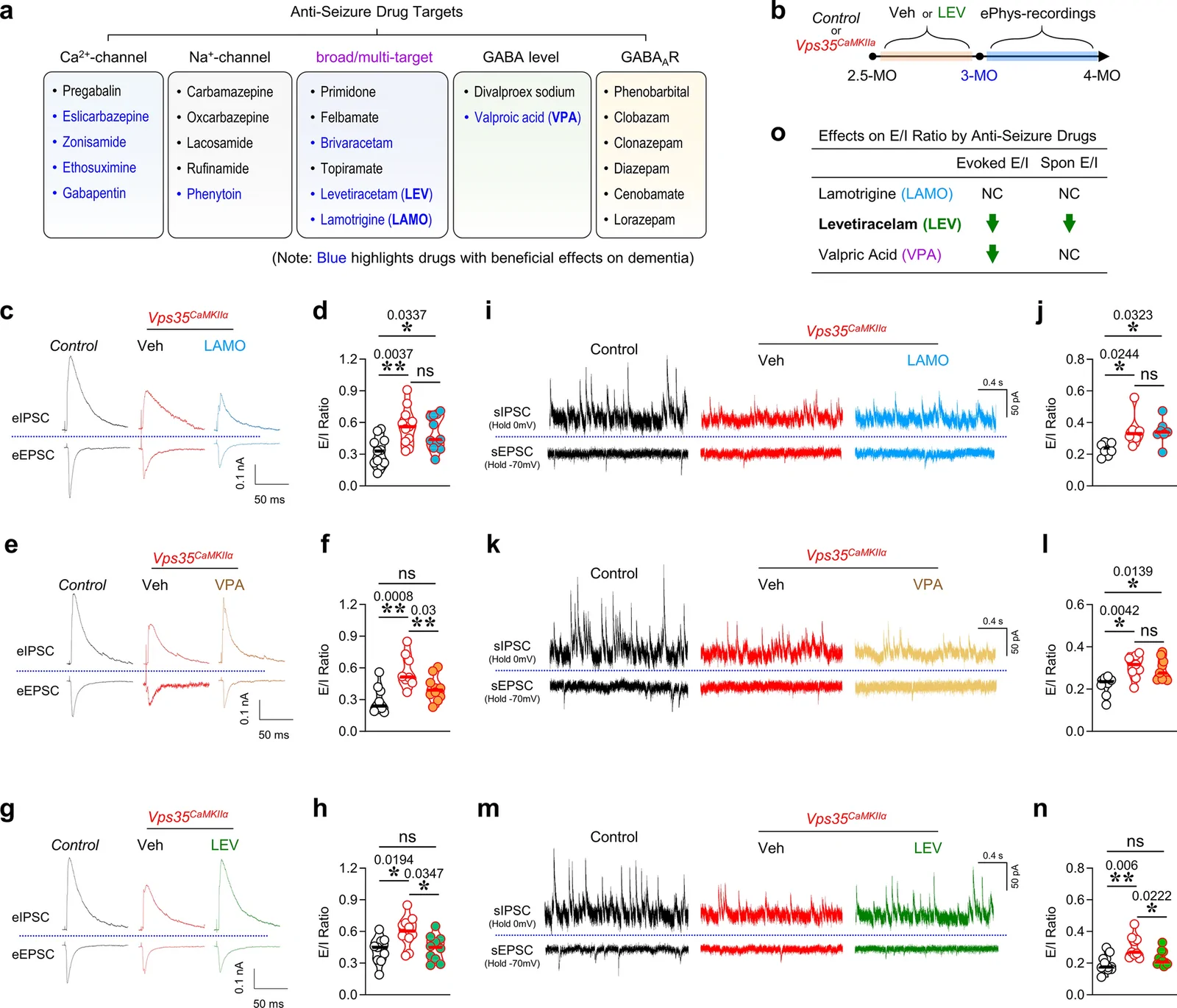
LEV selectively restores both evoked and spontaneous E/I balance. **a** Schematic overview of anti-seizure drug classes categorized according to their primary molecular targets, including Ca^2+^ channel modulators, Na^+^ channel blockers, drugs with mixed or multi-target mechanisms, and agents affecting GABAergic transmission. Drugs highlighted in blue indicate compounds previously reported to exhibit beneficial effects on cognition or dementia-related phenotypes. **b** Experimental timeline illustrating chronic LEV treatment followed by electrophysiological recordings and behavioral analyses. **c–h** Representative eEPSC and eIPSC traces recorded from CA1 pyramidal neurons in control mice and vehicle- or lamotrigine (LAMO)-treated (**c**), valproic acid (VPA)-treated (**e**), or levetiracetam (LEV)-treated (**g**) *Vps35*^*CaMKIIa*^ mice, and quantification of the corresponding evoked EPSC/IPSC ratios (**d**, **f**, and **h**). **i-n** Representative traces of spontaneous inhibitory postsynaptic currents (sIPSCs; holding potential, 0 mV) and spontaneous excitatory postsynaptic currents (sEPSCs; holding potential, −70 mV) recorded from CA1 pyramidal neurons in control mice and vehicle- or LAMO-treated (**i**), VPA-treated (**k**), or LEV-treated (**m**) *Vps35*^*CaMKIIa*^ mice, and quantification of the corresponding spontaneous E/I ratios (**j**, **l**, and **n**). All quantifications are presented as mean ± SD. Each dot represents one neuron (*n* = 10–12 neurons per group, obtained from at least four mice per genotype). Evoked and spontaneous E/I ratios were analyzed using one-way ANOVA followed by Tukey’s multiple comparisons test. *P < 0.05; **P < 0.01; ns, not significant. **o** Summary of the effects of anti-seizure drugs on evoked and spontaneous E/I balance in *Vps35*^*CaMKIIa*^ mice

Our results led to a hypothesis that restoring the E/I balance may be critical for alleviating cognitive deficits in *Vps35* mutant mice. To test this, we screened AEDs previously reported to benefit dementia patients, for their potential to modulate the E/I balance. From several classes of anti-seizure medications (Fig. 4a), we selected three compounds, LAMO, LEV, and VPA, for detailed analysis of their effects on E/I ratios in CA1 neurons of 3-MO *Vps35*^*CaMKIIα*^ mice. These AED compounds were chosen based on the following reasons: (1) previous reports indicate their cognitive benefits [27, 28, 84]; and (2) While SV2A-mediated excitatory neuronal synaptic transmission is believed to underscore LEV’s function [85, 86], whether and how LEV regulates neuronal E/I balance remain elusive [85, 87].

We examined the effects of these compounds by in vivo administration as illustrated in Fig. 4b. Specifically, beginning at 2.5 months of age, *Vps35*^*CaMKIIα*^ mice received daily i.p. injections of each compound for two weeks or more than two weeks (see method), followed by slice recordings at ≥3-MO. Notably, both LEV and VPA, but not LAMO, significantly suppressed the elevated E/I ratio in CA1 neurons (Fig. 4c−h), in which the E/I ratio was recorded by stimulating the SC-to-CA1 pathway, also named as evoked E/I as illustrated in Fig. 3a. We then examined the effects of these drugs on spontaneous E/I ratio in mutant CA1 neurons, in which the spontaneous inhibitory and excitatory postsynaptic currents (sEPSCs and sIPSCs) were recorded (Fig. 4i–n). LAMO and VPA treatment did not significantly change the spontaneous E/I ratio in the *Vps35*^*CaMKIIα*^ CA1 neurons (Fig. 4i–l). In contrast, LEV treatment significantly reduced the elevated spontaneous E/I ratio (Fig. 4m, n), indicating a broader effect on basal synaptic balance. Together, these data (Fig. 4o) demonstrate that anti-seizure drugs exert distinct and non-equivalent effects on the synaptic E/I balance in *Vps35*^*CaMKIIα*^ neurons. LAMO does not measurably alter evoked or spontaneous E/I ratios, whereas LEV modifies both evoked and spontaneous synaptic balance. VPA reduces evoked E/I responses without normalizing spontaneous synaptic activity (Fig. 4o).

**Fig. 5:**
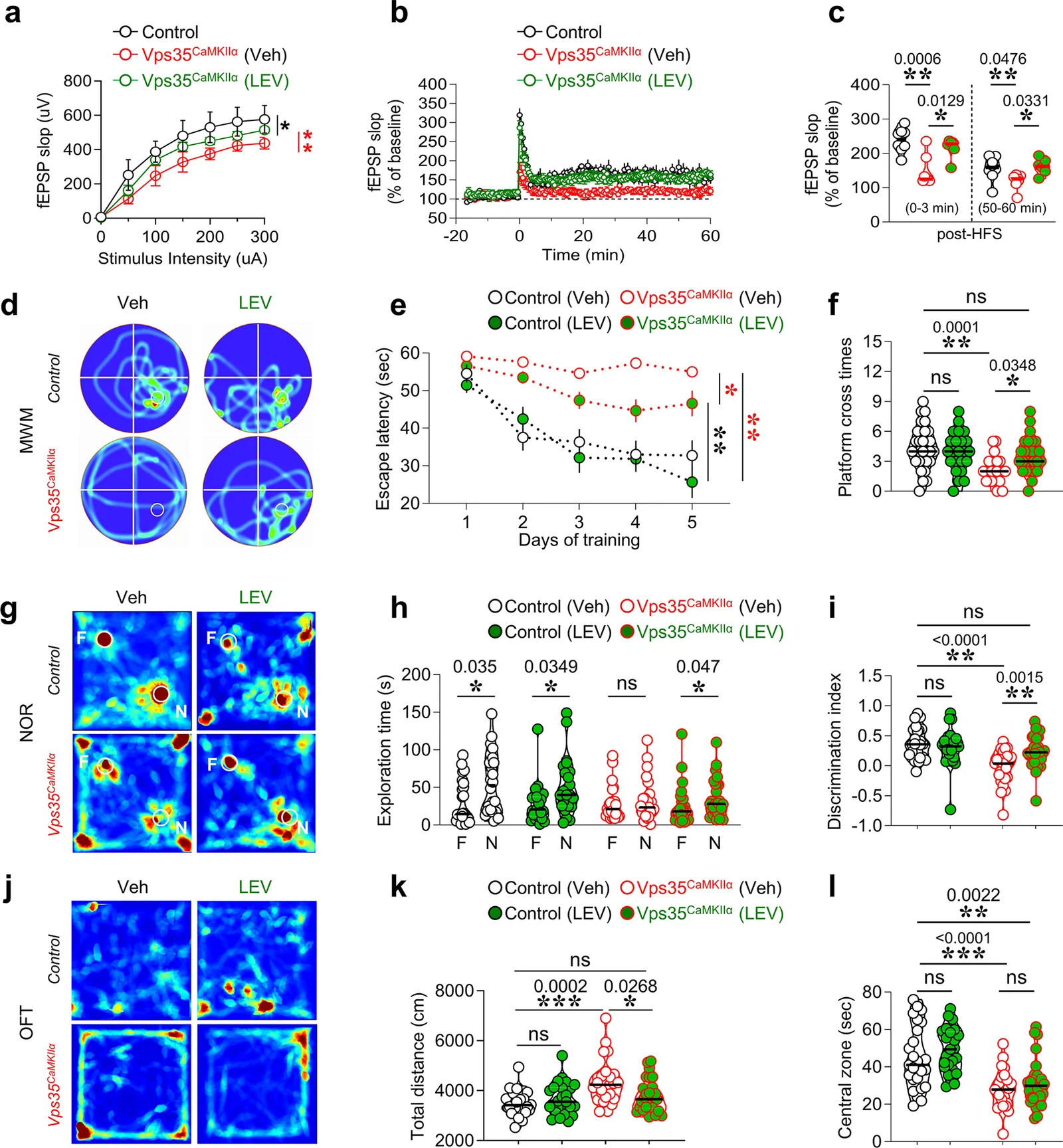
LEV improves synaptic plasticity and cognitive performance. **a** Input–output relationship of fEPSPs recorded at Schaffer collateral–CA1 synapses in control mice and *Vps35*^*CaMKIIa*^ mice treated with vehicle (Veh) or LEV. **b** Time course of fEPSP slopes recorded before and after high-frequency stimulation (HFS) at Schaffer collateral–CA1 synapses in control and *Vps35*^*CaMKIIa*^ mice treated with Veh or LEV. **c** Quantification of fEPSP slopes normalized to baseline during the early (0–3 min) and late (50–60 min) phases following HFS. **d–f** Representative heat maps from the Morris water maze (MWM) probe trial (**d**), escape latency during MWM training (**e**), and platform crossings during the MWM probe trial (**f**). **g–i** Novel object recognition (NOR) test. Representative exploration heat maps (**g**), quantification of exploration time for familiar (F) and novel (N) objects (**h**), and discrimination index (**i**). **j–l** Open field test (OFT). Representative locomotor activity heat maps (**j**), total distance traveled (**k**), and time spent in the center zone (**l**). All quantifications are presented as mean ± SD. Each dot represents one slice or one mouse, as indicated. Input–output relationships, LTP time courses, and escape latency during MWM training were analyzed using mixed-effects models (REML) followed by Sidak’s multiple comparisons test. Normalized fEPSP slopes during the early (0–3 min) and late (50–60 min) phases following HFS were analyzed using one-way ANOVA followed by Tukey’s multiple comparisons test. All other comparisons were analyzed using two-way ANOVA followed by Tukey’s multiple comparisons test. *P < 0.05; **P < 0.01; ***P < 0.001; ns, not significant

The superior normalization of both spontaneous and evoked E/I balance by LEV suggests that the SV2A-mediated modulation of synaptic vesicle release may be more effectively addressing the VPS35-related endosomal trafficking and presynaptic dysfunction than the broader mechanisms of VPA including GABA enhancement. We thus further examined whether LEV treatment could mitigate synaptic plasticity, LTP impairments and cognitive deficits in *Vps35*^*CaMKIIa*^ mice. LEV treatment significantly enhanced LTP following HFS of the SC-CA1 hippocampal pathway (Fig. 5a–c). In behavioral assessments, LEV improved learning and memory performance in both the MWM (Fig. 5d–f) and NOR tests (Fig. 5g–i), compared to the vehicle treatment. However, LEV did not alter the anxiety-like behavior, as reflected by the center zone time, despite a moderate reduction in overall locomotor activity in the OFT (Fig. 5j–l). Such a selective improvement in hippocampal-dependent tasks (MWM and NOR), but not open field behavior, may suggest that LEV preferentially normalizes VPS35-related synaptic dysfunction in cognitive circuits rather than broadly affecting non-cognitive behavioral domains.

Collectively, these results demonstrate that LEV effectively ameliorates the E/I imbalance and LTP deficits, leading to improved cognitive function in *Vps35*^*CaMKIIa*^ mice.

### LEV suppresses CA1 hyperexcitability, but has little effect on Aβ level, in *Vps35*^*CaMKIIa*^ mice by modulating synaptic input rather than intrinsic excitability

In order to understand how LEV rescues the E/I balance in *Vps35* mutant neurons, we began by assessing its effects on action potential firing of CA1 PyNs from 3-MO control and *Vps35*^*CaMKIIa*^ mice, given that LEV is a well-established AED, and neuronal excitability plays a central role in E/I balance [28, 85, 88]. LEV treatment significantly reduced action potential firing frequency in both control and mutant CA1 neurons at current injections < 250 pA (Fig. S9a, b). This reduction was accompanied by a slight decrease in input resistance in control animals, while RMPs remained largely unchanged across genotypes (Fig. S9c). The latter contrasts with the electrophysiological changes observed following Kir_2.1_ expression, which induced hyperpolarization of RMP and reduced hyperexcitability, but had little effect on the E/I imbalance in the mutant CA1 neurons (Fig. S7d–j). Moreover, while Aβ_42_ was elevated in *Vps35* mutant hippocampus, LEV treatments had little to no effect on it (Fig. S9d). Together, these results suggest that LEV suppresses neuronal hyperexcitability and restores E/I balance, which may be critical for its improvement of cognition, and may be primarily through changing the inputs to the mutant CA1 neurons rather than directly altering the intrinsic neuronal firing property or reducing Aβ levels.

### Impairments in glutamatergic transmission in *Vps35*^*CaMKIIa*^ CA1 neurons are unaffected by LEV treatment

**Fig. 6:**
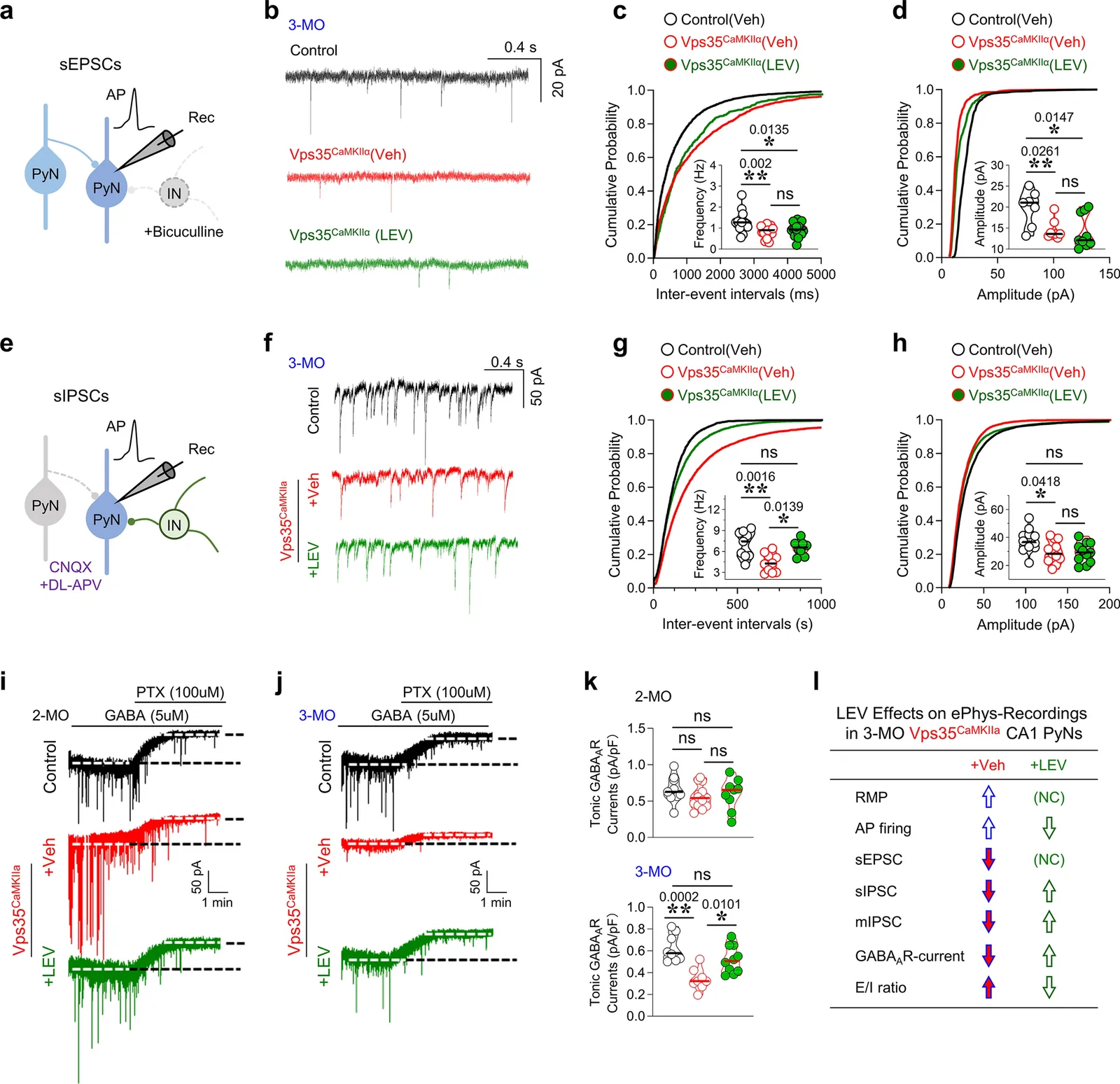
LEV enhances inhibitory synaptic transmission and GABA_A_ receptor-mediated currents. **a** Schematic illustration of whole-cell voltage-clamp recordings of spontaneous excitatory postsynaptic currents (sEPSCs) from CA1 pyramidal neurons (PyNs) in hippocampal slices in the presence of bicuculline. **b–d** Representative sEPSC traces recorded from 3-MO control mice and vehicle- or LEV-treated *Vps35*^*CaMKIIa*^ mice (**b**). Cumulative probability distributions of sEPSC inter-event intervals (**c**) and amplitudes (**d**) and insets show quantifications respectively. **e**. Schematic illustration of whole-cell voltage-clamp recordings of spontaneous inhibitory postsynaptic currents (sIPSCs) from CA1 pyramidal neurons in the presence of CNQX and DL-APV. **f–h** Representative sIPSC traces recorded from CA1 pyramidal neurons in 3-MO control and vehicle- or LEV-treated *Vps35*^*CaMKIIa*^ mice (**f**). Cumulative probability distributions of sIPSC inter-event intervals (**g**) and amplitudes (**h**) and insets show quantifications respectively. **i, j** Representative tonic GABA_A_ receptor-mediated currents recorded from CA1 pyramidal neurons in control mice and vehicle- or LEV-treated *Vps35*^*CaMKIIa*^ mice at 2-MO (**i**) and 3-MO (**j**). Tonic currents were revealed by bath application of GABA (5 μM) and blocked by picrotoxin (PTX, 100 μM). **k** Quantification of tonic GABA_A_ receptor-mediated currents at 2-MO and 3-MO. **l** Summary of LEV-induced changes in intrinsic excitability, excitatory and inhibitory synaptic transmission, GABA_A_ receptor-mediated currents, and E/I balance in CA1 pyramidal neurons of 3-MO *Vps35*^*CaMKIIa*^ mice. All quantifications are presented as mean ± SD. Each dot represents one neuron (*n* = 10–12 neurons per group, obtained from at least four mice per genotype). Cumulative distributions of sEPSC and sIPSC inter-event intervals and amplitudes were analyzed using the Kolmogorov–Smirnov test. Mean sEPSC and sIPSC frequency and amplitude, as well as tonic GABA_A_ receptor-mediated currents, were analyzed using one-way ANOVA followed by Tukey’s multiple comparisons test. *P < 0.05; **P < 0.01; ns, not significant

Given that the E/I balance relies on the dynamic regulation of both excitatory and inhibitory synaptic transmission, two key inputs to the CA1 neurons [89–92], we further analyzed sEPSCs in 3-MO *Vps35*^*CaMKIIa*^ mice, with or without LEV treatment. The sEPSCs were recorded in the presence of bicuculline to block GABAergic transmission and isolate excitatory inputs (Fig. 6a). CA1 neurons from *Vps35*^*CaMKIIa*^ mice exhibited significantly reduced sEPSC amplitude and frequency (Fig. 6b–d), consistent with previous reports of impaired AMPAR/NMDAR-mediated glutamatergic transmission and a reduced number of excitatory synapses in *Vps35*-deficient hippocampal neurons [38, 53, 93]. Notably, LEV treatment had minimal to no effect on sEPSC properties (Fig. 6b–d), suggesting that the therapeutic effect of LEV in *Vps35* mutant mice is unlikely to involve modulation of excitatory synaptic transmission.

### LEV restores GABAergic transmission in 3-MO *Vps35*^*CaMKIIα*^ CA1 PyNs

We then analyzed sIPSCs in CA1 PyNs from 3-MO control and *Vps35*^*CaMKIIa*^ mice, with or without LEV treatment. Recordings were performed in the presence of CNQX and DL-APV to block glutamatergic transmission (Fig. 6e). In 3-MO *Vps35* mutant CA1 neurons, both the frequency and the amplitude of sIPSCs were significantly reduced compared to controls (Fig. 6f–h), Intriguingly, LEV treatment increased the sIPSC frequency, but not amplitude, in mutant neurons (Fig. 6f–h). These results suggest that both PyN-VPS35 and LEV may play a role in maintaining the E/I balance by enhancing presynaptic GABA release and/or promoting the maturation or maintenance of inhibitory synapses.

To distinguish above possibilities, we recorded sIPSCs in CA1 PyNs from 2-MO control and mutant mice, an age at which the E/I balance remains intact in the mutants (Fig. 3b, c). In contrast to the deficits observed at 3 months, 2-MO *Vps35* mutant neurons showed an increased sIPSC frequency with no change in amplitude (Fig. S10a–c), arguing against a role for VPS35 in adult CA1 neurons to be required for GABAergic synapse formation/maturation. Instead, these findings suggest that VPS35 is necessary for the maintenance of the inhibitory synapses for homeostatic regulation.

This elevated sIPSC frequency may reflect enhanced pre-synaptic GABA release, increased number of GABAergic synapses, or higher neuronal firing rates. To distinguish among these possibilities, we measured PPRs of evoked IPSCs (eIPSCs) in 2- and 3-MO mice, with or without LEV treatment, to evaluate presynaptic GABA release probability. PPRs were comparable across all groups (Fig. S10d–g), suggesting that neither *Vps35* deletion nor LEV treatment significantly alters pre-synaptic release probability.

To further determine whether there are any alterations in pre- and post-synaptic GABAergic components, we assessed mIPSCs in CA1 neurons from 2- and 3-MO mice, with or without LEV treatment. In 2-MO mutants, the mIPSCs appeared to be normal (Fig. S11a–c); however, in 3-MO mutants, reduced frequency and amplitude in mIPSCs were notable, which were elevated by LEV treatment (Fig. S11d–f). Given the normal presynaptic GABA release probability in 3-MO mutant CA1 neurons, the reduced mIPSCs in 3-MO, but not 2-MO, suggest a progressive decline in the number of GABAnergic synapses.

Finally, we investigated the tonic GABAergic currents, which are primarily mediated by peri- and extra-synaptic GABA_A_ receptors. Thus, Picrotoxin (PTX), a GABA_A_ receptor channel blocker, was used to isolate the tonic current component. In 2-MO mutants, the tonic GABA_A_ receptor-mediated currents were comparable to controls (Fig. 6i, k), whereas a significant reduction emerged in 3-MO mutants (Fig. 6j, k). Notably, LEV treatment enhanced tonic GABA currents in 3-MO mutants, although not to control levels (Fig. 6f, h).

Collectively, these results (Fig. 6i) suggest that CA1 neurons in 2-MO *Vps35* mutant mice maintain the E/I balance, potentially through compensatory increases in GABAergic transmission. The E/I balance is disrupted in 3-MO mutant neurons, likely due to impaired GABAergic transmission at both presynaptic and postsynaptic levels. LEV treatments restore both evoked and spontaneous E/I balance, possibly by promoting the maintenance of inhibitory synaptic structures.

### Decreased density but increased size of GABAergic synapses in 3-MO *Vps35*^*CaMKIIa*^ mice are diminished by LEV treatment

**Fig. 7:**
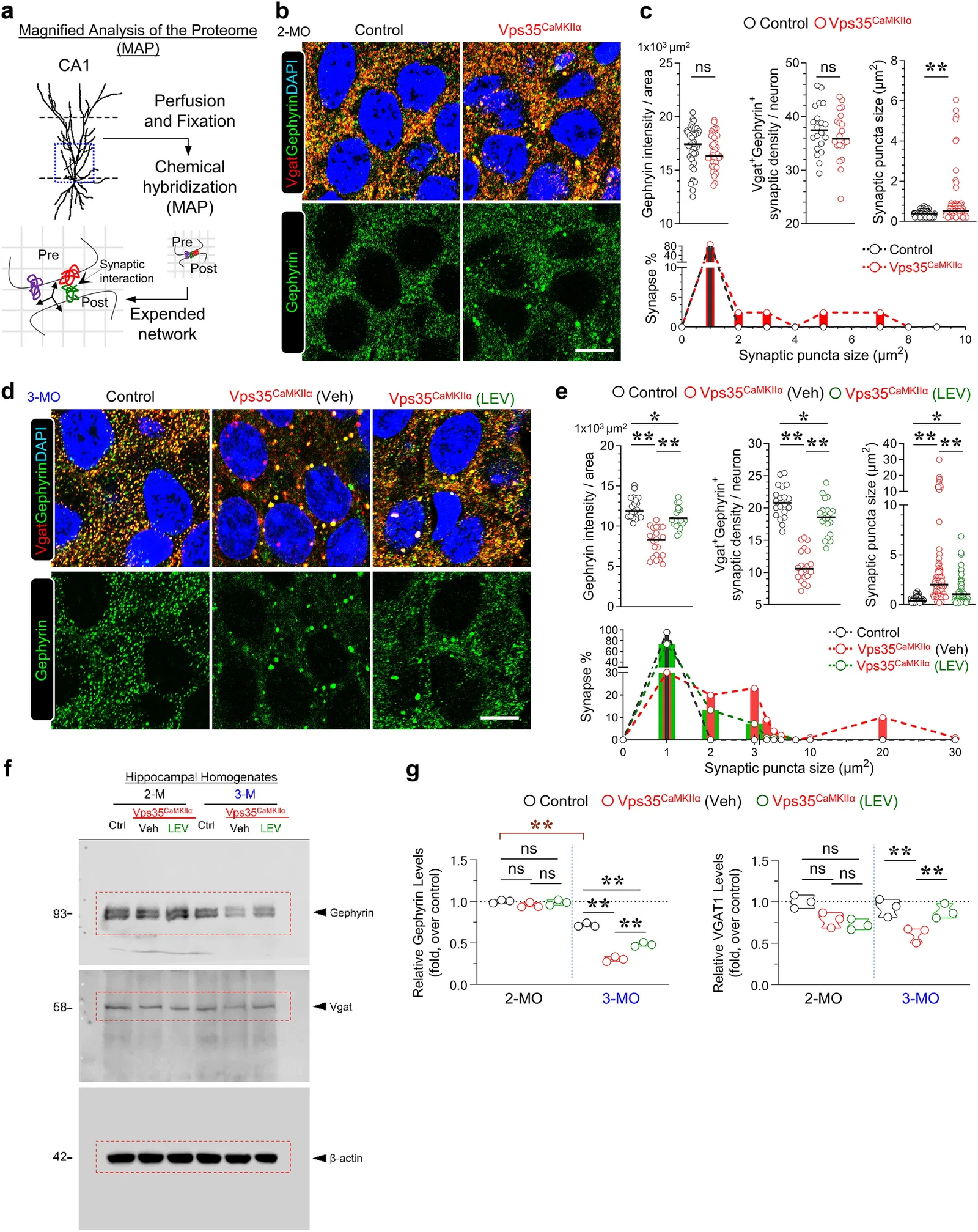
Reduced density but increased size of GABAergic synapses in 3-MO *Vps35*^*CaMKIIa*^ mice, which was normalized by LEV treatment. **a** Schematic of the magnified analysis of the proteome (MAP) experimental protocol. **b, c** Representative MAP co-immunostaining analysis of Vgat (red) and Gephyrin (green) in the CA1 stratum pyramidal (SP) region of 2-MO *Vps35*^*CaMKIIa*^ and control (**b**), and the quantifications (mean and appearance line at median, *n* = 40 from 3 mice per group, **P < 0.01, student’s *t*-test) are shown (**c**). **d, e** Representative co-immunostaining analysis of Vgat (red) and Gephyrin (green) in the CA1 SP region of the 3-MO *Vps35*^*CaMKIIa*^ injected with vehicle or LEV, or control mice (**d**), and the quantifications (mean and appearance line at median, *n* = 20–100 from 3 mice per group, **P < 0.01, student’s t-test or one-way ANOVA with Tukey's HSD test) are shown (**e**). **f, g** Representative Western blots of hippocampal homogenates from 2-MO and 3-MO mice with indicated conditions and antibodies (**f**), and quantifications (**g**) (mean ± SD, *n* = 4 independent experiments, *P < 0.05, **P < 0.01, one-way ANOVA test with Tukey's HSD test). The brown asterisk (*) indicates the comparison between the 2-MO and 3-MO groups. The black star (*) indicates a comparison between indicated experiment groups

To further test the above hypothesis, we employed expansion microscopy, a super-resolution fluorescence imaging technique that physically enlarges brain tissue by embedding it in a swellable polymer gel [94, 95]. This method, also called “magnified analysis of proteome” (MAP), enables nanoscale imaging of cellular structures such as synapses using conventional or confocal microscopes [96]. The brains of *Vps35*^*CaMKIIα*^ mice at 2- or 3-MO, with or without LEV treatments were collected, embedded in the expandable gel, and sectioned for co-immunostaining and MAP analysis (Fig. 7a).

To label inhibitory synapses, we used antibodies against Vgat, a presynaptic GABA transporter, and gephyrin, a postsynaptic scaffold protein that anchors GABA_A_ receptors [97–102]. In the CA1 region of *Vps35* mutant mice at 2-MO, the density of Vgat^+^/gephyrin^+^ synapses was largely comparable to those in controls (Fig. 7b, c). However, a small subset of Vgat^+^/gephyrin^+^ synapses was enlarged in size in the mutants (Fig. 7b, c). Considering the electrophysiological findings, these results suggest that the GABAergic synapses in mutant CA1 neurons are largely preserved at this stage, and the increased sIPSC frequency is likely due to the elevated neuronal firing in 2-MO mutant CA1 neurons.

Notably, the GABAergic synapses in 3-MO CA1 region showed a significant reduction in the density of Vgat^+^/gephyrin^+^ synapses in the mutants compared to controls (Fig. 7d, e). The remaining Vgat^+^/gephyrin^+^ synapses in the mutant CA1 were obviously enlarged, appearing to be protein aggregates (Fig. 7d, e). Strikingly, LEV treatment significantly restored the density of Vgat^+^/gephyrin^+^ synapses and reduced the enlarged Vgat^+^/gephyrin^+^ aggregates (Fig. 7d, e). These findings align with the electrophysiological recordings (Fig. 6f, g, j, k), underscoring the essential role of VPS35 and the therapeutic potential of LEV in maintaining GABAergic synapses in CA1 neurons.

Western blot analyses further supported these results, showing that Vgat and gephyrin protein levels were unchanged at 2-MO, but significantly reduced at 3-MO in mutant hippocampus, and partially restored by LEV treatment (Fig. 7f, g).

Additionally, MAP analysis of excitatory synapses using antibodies against Vglut1, a presynaptic glutamate transporter, and PSD95, a postsynaptic scaffold protein that anchors AMPA and NMDA receptors, showed significant reductions in Vglut1^+^/PSD95^+^ synaptic puncta and corresponding protein levels in 3-MO *Vps35* mutant hippocampus (Fig. S12), suggesting that VPS35 in CA1 neurons is necessary for the maintenance of excitatory synapses. However, the deficits in the excitatory synapses were not rescued by LEV treatment, although PSD95 puncta was increased by LEV (Fig. S12), consistent with the sEPSC recordings (Fig. 6b–d), indicating that LEV has minimal effect on excitatory synapses.

Collectively, these findings, in conjunction with electrophysiological data, provide strong support for the role of VPS35 in maintaining GABAergic synapse integrity in CA1 neurons and highlight a specific effect of LEV in restoring inhibitory, but not excitatory, synaptic deficits.

### Increases of SV2A, VPS26a, and GABAergic synaptic proteins in 3-MO *Vps35*^*CaMKIIa*^ hippocampus by LEV treatment

**Fig. 8:**
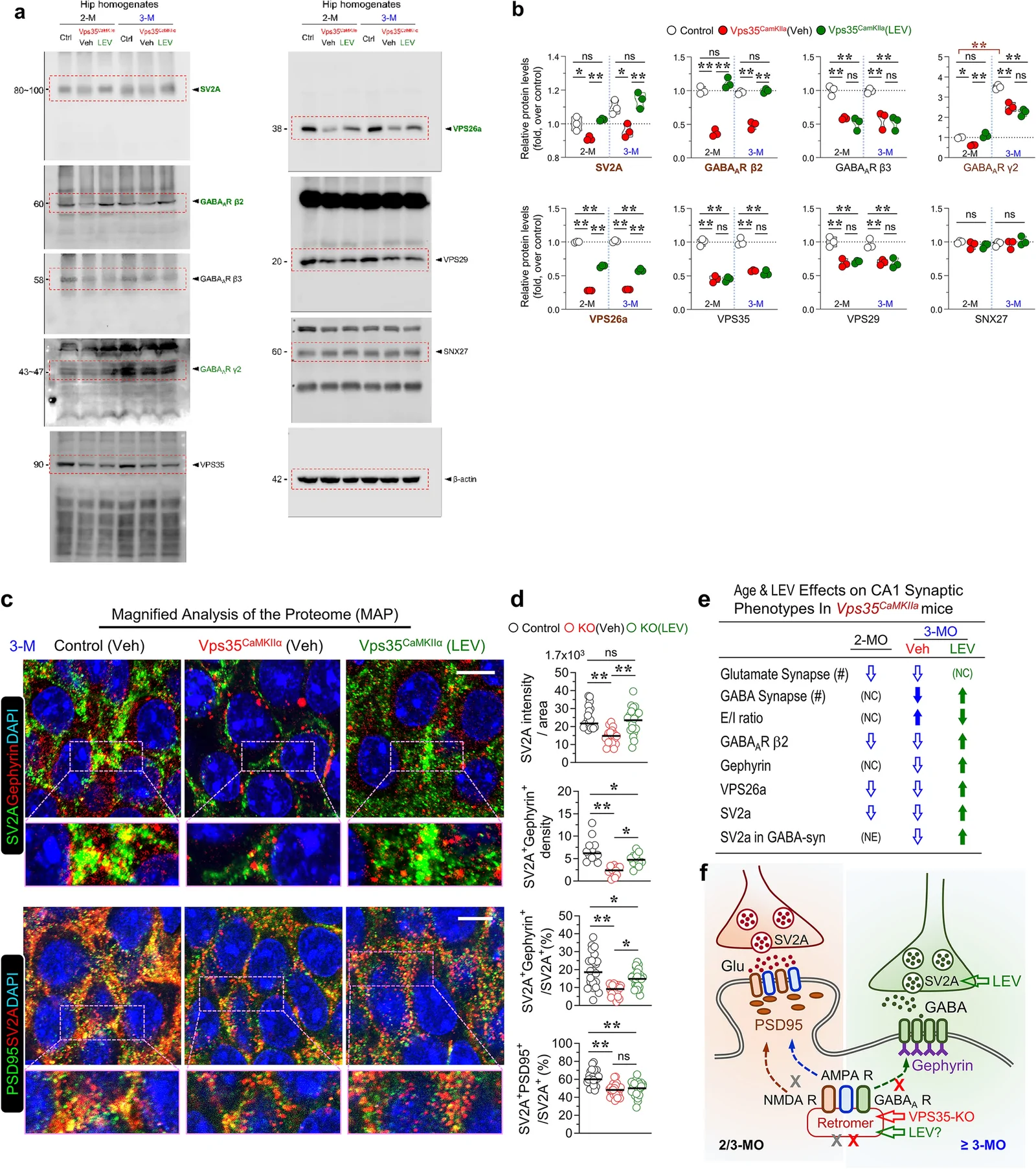
LEV restoration of SV2A, VPS26a, and GABAergic synaptic proteins in 3-MO *Vps35*^*CaMKIIα*^ hippocampus. **a, b** Representative Western blots of hippocampus homogenates from 2-MO and 3-MO mice with indicated conditions and antibodies (**a**) and quantifications (**b**) (mean ± SD, *n* = 3 independent experiments, **P < 0.01, one-way ANOVA test with Tukey's HSD test). The brown asterisk (*) indicates the comparison between the 2-M and 3-M groups. The black star (*) indicates a comparison between indicated experiment groups. **c, d** Representative MAP co-immunostaining analysis of SV2A (green) and Gephyrin (red) in the CA1 stratum pyramidal (SP) region of 3-MO mice with indicated conditions (**c**, upper), MAP co-immunostaining analysis of PSD95 (green) and SV2A (red) (**c**, bottom), and the quantifications (mean and appearance line at median, *n* = 40 from 3 mice per group, *P < 0.05, one-way ANOVA test with Tukey's HSD test) (**d**). **e** Summary of age & LEV effects on CA1 synaptic phenotypes in *Vps35*^*CaMKIIa*^ mice. **f** The schematic of a working model in which VPS35 maintains GABAergic synaptic proteins in an age-dependent manner, while LEV selectively normalizes multiple synaptic proteins, including SV2A and VPS26a at inhibitory synapses

To understand how VPS35 and LEV preserve GABAergic synapses, we first examined the expression levels of postsynaptic GABAergic proteins, retromer complex components, and SV2A, a principal binding target of LEV and a synaptic vesicle protein [85, 88] in 2- and 3-MO hippocampus, with or without LEV treatment. Western blot analyses revealed reduced levels of several GABA_A_ receptor subunits (β2, β3, and γ2), SV2A, and the retromer proteins VPS26a and VPS29 in the hippocampus of both 2- and 3-MO mutants (Fig. 8a, b). Interestingly, among these proteins, LEV treatment increased the protein levels of GABA_A_R β2, SV2A, and VPS26a in 2- and 3-MO mutants (Fig. 8a, b).

We next asked whether the LEV-induced increase in SV2A was associated with inhibitory or excitatory synapses. Co-immunostaining of expansion microscopy brain sections using antibodies against SV2A and gephyrin (inhibitory synapses) or PSD95 (excitatory synapses) showed that approximately 20% of SV2A-positive vesicles co-localized to gephyrin^+^ inhibitory synapses in the CA1 region of 3-MO control mice, with ~60% primarily associated with excitatory synapses (Fig. 8c, d). While SV2A levels were markedly reduced in *Vps35* mutants, LEV selectively restored SV2A abundance at gephyrin^+^ inhibitory synapses, but not at PSD95^+^ excitatory synapses (Fig. 8c, d). These findings (Fig. 8e, f) suggest that VPS35 maintains GABAergic synaptic proteins in a progressive manner, and LEV may normalize multiple synaptic proteins, including SV2A, selectively at inhibitory synapses.

## Discussion

VPS35 dysfunction is recognized as a risk factor for several neurodegenerative diseases, including AD and PD [38, 41, 47]. In this study, we demonstrate that VPS35 loss in postnatal PyNs of the hippocampus and cortex leads to a progressive decline in cognitive functions relevant to AD and ADRD. These behavioral impairments are closely associated with an imbalance in E/I neurotransmission in CA1 PyNs, independent of the depolarized RMP-driven hyperexcitability. Further analyses revealed that the E/I imbalance in mutant CA1 neurons arises primarily from deficits in GABAergic transmission, affecting both pre-GABAergic synaptic inputs/number and post-synaptic GABA_A_ receptor levels. Intriguingly, chronic administration of LEV effectively restored the E/I balance and improved the cognition in the mutant mice, likely by enhancing or preserving GABAergic synaptic function. Together, these findings (summarized in Fig. 8e) identify a previously unrecognized role for VPS35 in maintaining GABAergic transmission and E/I balance in hippocampal circuits. They establish a mechanistic link of impaired inhibitory synaptic function and E/I balance with cognitive decline and suggest that LEV may hold therapeutic potential in AD/ADRD by stabilizing inhibitory synaptic proteins and promoting network integrity.

Hyperexcitability, defined as abnormal increases in neuronal activity or firing, is increasingly recognized as an early indicator of neuropathology in various dementias, including AD and ADRD. Multiple lines of evidence support a potential link between hyperexcitability and disease progression. First, EEG studies have shown increased epileptiform activity in patients with mild cognitive impairment or AD compared to age-matched controls [19, 20, 103, 104]. Second, transgenic AD mouse models, such as those expressing the Swedish mutant human *APP* gene, exhibit hippocampal hyperexcitability before the onset of amyloid plaque deposition and cognitive decline [24, 72]. Third, certain AEDs, including LEV, have demonstrated potential to improve memory in mild cognitive impairment patients exhibiting signs of hyperexcitability [28, 84]. Despite these findings, the precise role of hyperexcitability in AD/ADRD pathogenesis, and the mechanisms by which LEV and other AEDs may confer cognitive benefits, remain elusive. Key questions of ongoing interest include (1) how AD risk factors drive neuronal hyperexcitability, (2) in what ways hyperexcitability contributes to cognitive dysfunction, and (3) how AEDs like LEV ameliorate these effects in the context of AD and ADRD.

Here, our studies show that deficiency of VPS35, a genetic risk factor for AD/ADRD, in excitatory neurons induces hyperexcitability through at least two distinct mechanisms. First, VPS35 loss alters RMP, likely due to the downregulation of potassium channels, such as the Kir_2.1,_ thereby lowering the threshold for neuronal firing (Fig. 2j and Fig. S5b–g). Second, VPS35 deficiency impairs GABAergic inhibitory transmission, likely resulting in an increased E/I ratio (Fig. 3a–c). Interestingly, while Kir_2.1_ expression prevented hyperexcitability and induced hypoexcitability in CA1 neurons, it exerted only a minimal impact on E/I imbalance and cognitive performance in *Vps35*^*CaMKIIα*^ mice (Figs. S7–S8). In contrast, LEV effectively normalized E/I balance and significantly improved cognitive function in *Vps35*^*CaMKIIα*^ mice, likely by restoring or preserving the GABAergic transmission (Fig. 4g, h, m, n; Fig. 5, and Fig. 6). These findings thus suggest that the impaired inhibitory signaling/inputs, rather than increased intrinsic excitability alone, may play a more central role in cognitive decline associated with VPS35 deficiency and possibly broader AD/ADRD pathophysiology.

It should be noted that at 2-MO, the VPS35-deficient CA1 neurons exhibited hyperexcitability, but their E/I ratio remained comparable to that of controls (Figs. 2g–k and Fig. 3b, c). By 3-MO, the E/I transmission was imbalanced, accompanied by GABAergic transmission impairment (Figs. 3b, c and Fig. 6), implicating the importance of maintaining GABAergic transmission for E/I balance. These findings also raise the question of how VPS35 deficiency in excitatory neurons impairs GABAergic transmission in 3-MO, but not 2-MO, although the excitability remains high in both 2- and 3-MO mutant CA1 neurons (Fig. 2g–k). The preserved E/I balance in 2-MO mutant CA1 neurons appears to result from a compensatory increase in GABAergic transmission, as evidenced by elevated frequency of sIPSCs but normal mIPSCs (Fig. S10a–c and S11a–c), suggesting a homeostatic response to early hyperexcitability. This compensatory mechanism becomes maladaptive over time. By 3-MO, the VPS35-deficient neurons show reductions in mIPSCs (Fig. S11d–f), sIPSCs (Fig. 6e-h), as well as both inhibitory synapse density and postsynaptic GABA_A_ receptor expression (Fig. 7), leading to impaired inhibitory signaling, disruption of the E/I balance, and the emergence of neuropathological and behavioral deficits.

How does VPS35 in excitatory neurons play a role in maintaining GABAergic inputs/synapses? One explanation is the regulation of post-synaptic transmembrane proteins, such as Neuroligin2. Neuroligin2 is a cargo of retromer expressed in excitatory neurons and is crucial for maintaining inhibitory synapses via its trans-interaction with neurexins [105–107]. In addition, other retromer cargos, such as GABA receptors, are also expressed in excitatory neurons and play crucial roles in the formation and maintenance of postsynaptic GABAergic synapses [108, 109]. An age-dependent disruption of the trafficking or function of these cargos likely occurs in VPS35-deficient neurons, which may underlie the observed age-associated defects in GABAergic transmission and inhibitory synapses. Further investigation into the specific roles of these retromer cargos in inhibitory synapse integrity, transmission, and E/I balance could yield valuable insight into the mechanisms by which VPS35 deficiency contributes to AD/ADRD pathogenesis.

It is noteworthy that prior studies have shown that the network hyperexcitability can arise from reduction of Nav1.1-dependent function in PV interneurons [25, 110], as well as from postsynaptic chloride dyshomeostasis and impaired GABAergic inhibition [111, 112]. While the present work suggests that *Vps35* downregulation may represent an upstream mechanism that contributes to inhibitory circuit dysfunction, altered neural network activity, and Aβ accumulation, it would be of interest to investigate whether PV interneurons and postsynaptic chloride homeostasis are also impaired in *Vps35*-deficit brains in future.

Also noteworthy is that our observation of increased Aβ_42_ levels in *Vps35* mutant hippocampus (Fig. S9d) is in line with previous reports [46]; however, they do not support the idea that hyperexcitability in VPS35-deficient mice drives the Aβ increase, given that LEV suppressed hyperexcitability but had little effect on Aβ levels (Fig. S9d).

In addition, our findings support the notion that hyper-active or E/I-imbalanced neurons can disrupt the synchrony and temporal precision of brain networks critical for learning and memory, such as the hippocampus and entorhinal cortex. This disruption likely impairs synaptic plasticity (e.g., LTP), a fundamental mechanism for encoding new memories. It is also important to consider that chronic hyperexcitability may increase the metabolic demand and lead to excitotoxicity caused by excessive glutamatergic activity, which in turn promotes neuronal death. This can accelerate neurodegeneration and perpetuate a vicious cycle of cognitive decline and network hyperactivity. Although our analyses in 2–3-MO mutant mice revealed little to no changes in neuronal density, further investigation into neuronal morphology, cell loss, and markers of neurodegeneration in older animals will be critical for understanding the long-term impact of VPS35 deficiency or hyperexcitability on brain structure and function.

Furthermore, our studies have demonstrated the possible therapeutic potential of LEV in *Vps35*-deficiency-associated AD/ADRD. The beneficial effects of LEV are supported by its function in promoting the E/I balance, suppressing hyperexcitability, and increasing LTP and cognitive function in 3-MO *Vps35*^*CaMKIIa*^ mice. This LEV effect requires chronic treatment. Our findings in *Vps35*-deficient animal models are consistent with previous clinical reports. In a randomized clinical trial, while LEV did not improve executive function as measured by NIH executive ability scores, it did improve the performance in spatial memory and cognitive tasks and epileptiform activity in patients with AD [28, 84, 113, 114].

Regarding the effects on cognitive performance, LEV treatment resulted in a partial rather than full rescue, suggesting additional mechanisms by which VPS35 regulates cognitive function. With respect to the mechanisms underlying the effect of LEV on inhibitory synapses and transmission, we propose that by maintaining GABAergic inhibitory input to excitatory neurons, LEV may prevent hyperexcitability, promote the E/I balance within hippocampal neural networks, and improve cognitive function. This mechanism may also underlie, at least in part, the well-established anti-seizure effects of LEV. However, how LEV selectively improves inhibitory (but not excitatory) synapses is an important question. Although SV2A is a high-affinity binding target of LEV [85], and likely plays a role in stabilizing presynaptic components, its ubiquitous expression in both excitatory and inhibitory neurons [115, 116] (Fig. 8) complicates the notion that SV2A alone accounts for the selective action of LEV on GABAergic synapses. We speculate that the preferential effect of LEV on GABAergic synapses may instead be mediated through its stabilization of a broader set of GABAergic synaptic proteins. These include the presynaptic vesicular GABA transporter Vgat, as well as key postsynaptic scaffolding and receptor proteins such as gephyrin and GABA_A_ receptor β2 subunit (GABA_A_Rβ2). This hypothesis is supported by our observations (Figs. 7 and 8) and consistent with recent proteomic analyses [117].

Given that multiple proteins, particularly GABAergic synaptic proteins, are upregulated following chronic LEV treatment under pathological conditions (e.g., nVPS35-KO mice Figs. 7 and 8 and AD mouse models [28, 117]), it is possible that LEV may influence protein trafficking and degradation via the endosomal–lysosomal pathway. Supporting this notion, LEV chronic treatment in an AD mouse model has been shown to normalize the levels of several key proteins involved in the endocytic–lysosomal machinery, including AP-2, dynamin 1, synaptotagmin, and v-ATPase. These findings imply that LEV may enhance impaired synaptic vesicle recycling in disease states [117].

Interestingly, among the proteins with expression normalized by LEV in AD models, many are retromer cargos (e.g., GABA_A_Rβ2, v-ATPase, neuroligin 2) and components of the retromer complex itself (e.g., VPS26, sorting nexins) [117]. Although no direct binding between LEV and retromer complex proteins has been reported, the functional overlap between LEV and VPS35/retromer is notable. Both are implicated in regulating endosomal protein trafficking and APP cleavage, preventing neuronal hyperexcitability, maintaining E/I balance, and supporting cognitive functions (Figs. 4g–h, m–n,; Fig. 5a–i; Figs. 6, 7 and 8; Figs. S9 and S10) [28, 84, 117–119]. These parallels raise the intriguing possibility that components of the retromer complex may represent direct or indirect molecular targets of LEV, a hypothesis that warrants further investigation.

## Conclusion

Within this study, our results suggest that the E/I imbalance-associated hyperexcitability is both a marker and a potential driver of cognitive decline in *Vps35*-deficient mice. LEV appears to be a potential therapeutic strategy for ADRDs through preservation of GABAergic synapses and E/I balance. Restoring the E/I balance may therefore represent a viable therapeutic strategy to delay or prevent cognitive deterioration.

## Supplementary Information

Additional file 1. **Fig. S1**. Validation of conditional Vps35 deletion and normal gross physiological and hippocampal features. **Fig. S2**. No positional or object exploration bias in 3-MO *Vps35*^*CaMKIIα*^ mice before NOR testing. **Fig. S3**. Normal anxiety-related behaviors and cognitive functions in 2-MO littermate control and *Vps35*^*CaMKIIa*^ mice. **Fig. S4**. Impaired learning and memory in 3-MO *VPS35*^*CaMKIIa*^ female mice. **Fig. S5**. Decreased Kir2.1 expression and function in hippocampal pyramidal neurons. **Fig. S6**. Adult-onset hippocampal Vps35 deletion recapitulates CA1 neuronal hyperexcitability, E/I imbalance, and impaired recognition memory. **Fig. S7**. Restored Kir2.1 relieved seizure susceptibility and decreased CA1 neuronal hyper-excitability. **Fig. S8**. Kir2.1 restoration was insufficient to rescue learning and memory in *Vps35*^*CaMKIIa*^. **Fig. S9**. Levetiracetam attenuates intrinsic excitability of CA1 pyramidal neurons. **Fig. S10**. LEV rescues deficits in inhibitory synaptic transmission. **Fig. S11**. LEV rescues deficits in miniature inhibitory synaptic transmission. **Fig. S12**. Age-dependent excitatory synapse impairment in the CA1 hippocampus of *Vps35*^*CaMKIIα*^ mice.

Additional file 2. Uncropped blots.

## Data Availability

No datasets were generated or analysed during the current study.

## Abbreviations

ACSF: Artificial cerebrospinal fluid
AEDs: Anti-epileptic drugs
E/I: Excitatory/Inhibitory
EEG: Electroencephalography
EPM: Elevated plus maze
fEPSPs: Field excitatory postsynaptic potentials
FST: Forced swim test
Kir: Inwardly rectifying K^+^
LAMO: Lamotrigine
LEV: Levetiracetam
LTP: Long-term potentiation
mIPSCs: Miniature inhibitory postsynaptic currents
MWM: Morris water maze
NOR: Novel object recognition
OFT: Open field test
PPR: Paired-pulse ratio
PTX: Picrotoxin
PyNs: Pyramidal neurons
RMP: Resting membrane potential
sEPSCs: Spontaneous excitatory postsynaptic currents
sIPSC: Spontaneous inhibitory postsynaptic currents
SV2A: Synaptic vesicle glycoprotein 2a
TST: Tail suspension test
TTX: Tetrodotoxin
VPA: Valproic acid
VPS26A: Vacuolar protein sorting-associated protein 26A
VPS35: Vacuolar protein sorting-associated protein 35
