# Supplementary material for: Levetiracetam restores GABAergic function and network homeostasis following Vps35 deficiency in excitatory neurons
Source: Transl Neurodegener. 2026 Oct 14;15:54. doi: 10.1186/s40035-026-00585-x (PMC13662985; doi:10.1186/s40035-026-00585-x)
Supplement: Supplementary file 1 [file 40035_2026_585_MOESM2_ESM.docx]

Fig. 7f

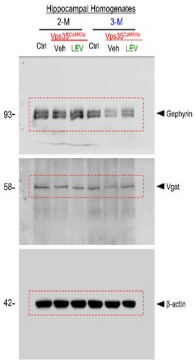
Hippocampal Homogenates
2-M
3-M
Vps35CaMKIIα
Vps35CaMKIIα
Ctrl
Veh
LEV
Ctrl
Veh
LEV
93-
◀ Gephyrin
58-
◀ Vgat
42-
◀ β-actin
