# Supplementary material for: Levetiracetam restores GABAergic function and network homeostasis following Vps35 deficiency in excitatory neurons
Source: Transl Neurodegener. 2026 Oct 14;15:54. doi: 10.1186/s40035-026-00585-x (PMC13662985; doi:10.1186/s40035-026-00585-x)
Supplement: Supplementary file 1 [file 40035_2026_585_MOESM1_ESM.docx]

**Supplementary Information**

**Levetiracetam restores GABAergic function and network homeostasis following Vps35 deficiency in excitatory neurons**

Zhi-Peng Liu^1,#^, Daehoon Lee^1,2,#^, Zhibing Tan^1^, Anika Wu^1^, Lin Mei^1,3^ and Wen-Cheng Xiong^1,2*^

^1^Department of Neurosciences, Case Western Reserve University, School of Medicine, Cleveland, Ohio, 44106, USA

^2^State Key Laboratory of Transvascular Implantation Devices, The Second Affiliated Hospital**,** Zhejiang University, Hangzhou 310009, China

^3^Chinese Institute of Medical Research, Capital Medical University, Beijing, 100069, China

^#^These authors contributed equally to this work

*Corresponding author

Wen-Cheng Xiong

Email: [Wen-Cheng.Xiong@case.edu](mailto:Wen-Cheng.Xiong@case.edu)

Phone: 216-368-4865

**Supplementary Figures**

**Fig. S1** Validation of conditional Vps35 deletion and normal gross physiological and hippocampal features. **a** Representative immunoblots showing protein levels of retromer components in hippocampal homogenates at 3 months of age, with β-actin as a loading control. **b** Quantification of VPS35, VPS26a, and VPS29 protein levels normalized to β-actin. **c** Representative RNAscope analysis of VPS35-mRNAs, which co-immunostained with NeuN, in the CA1 of control and Vps35*^CaMKIIa^***. d** Quantification of VPS35-mRNAs distribution and levels in NeuN^+^ or NeuN^-^ cells. **e** Body weight measured weekly in male mice over the first 12 weeks of postnatal development. **f** Representative coronal brain sections showing immunostaining for S100β and NeuN across cortical (Ctx) and hippocampal (CA1) regions. Boxed areas indicate regions shown at higher magnification. **g** Quantification of S100β fluorescence intensity in cortex and hippocampus. **h** Quantification of NeuN fluorescence intensity in cortex and hippocampus. **i** Representative immunofluorescence images showing GFAP and DCX staining in hippocampal sections. **j** Quantification of GFAP fluorescence intensity in the hilus and molecular layer (ML). **k** Quantification of DCX-positive cell density in the dentate gyrus (DG). All quantifications are presented as mean ± SD. Each dot represents one biological sample (Western blot), one mouse (body weight), or one image field (immunofluorescence), as indicated. Protein expression levels in hippocampal homogenates were analyzed using unpaired two-tailed Student’s t tests (n = 3 mice per group). Body weight data were analyzed using a mixed-effects model (REML), with time as a repeated factor and genotype as a between-subject factor (*n* = 28-30 mice, per genotype). Immunofluorescence quantifications were analyzed unpaired two-tailed Student’s *t*-test *n* = 3–5 image fields per region per mouse, obtained from at least three mice per genotype). **P* < 0.05; ***P* < 0.01; ns, not significant.

**Fig. S2** No positional or object exploration bias in 3-MO *Vps35^CaMKIIα^* mice before NOR testing. **a-b** Open field habituation session performed before the novel object recognition (NOR) task. Representative locomotor activity heat maps from 3-MO control and *Vps35^CaMKIIα^* mice during arena habituation (**a**), and quantification of time spent in each quadrant (Q1–Q4) during the habituation session (**b**). **c-d** NOR training phase with two identical objects. Representative exploration heat maps from control and Vps35^CaMKIIα^ mice during training (**c**). Identical objects (Obj1 and Obj2) are indicated. Quantifications of exploration time for the two identical objects (left) and object preference index (right) during the training session are shown in (**d**). All quantifications are presented as mean ± SD. Each dot represents one mouse (*n* = 30–40 mice per group). Time spent in individual quadrants during habituation and exploration time during the training session were analyzed using two-way ANOVA followed by Sidak’s multiple comparisons test. Object preference index was analyzed using unpaired two-tailed Student’s t-test. ns, not significant.

**Fig. S3** Normal anxiety-related behaviors and cognitive functions in 2-MO littermate control and *Vps35^CaMKIIa^* mice. **a** Representative heat maps of trajectories during the open field test (OFT). **b** Quantification of total distance traveled (left) and time spent in the central zone (right) during the OFT. **c** Representative traces from the elevated plus maze test (EPMT). **d** Quantification of time spent in the open arms (left) and number of open arm entries (right) during the EPMT. **e** Representative heat maps from the Morris water maze (MWM) probe trial. **f** Escape latency during MWM training across consecutive training days (left) and quantification of platform crossings during the probe trial (right). **g** Representative exploration heat maps from the novel object recognition (NOR) test. Familiar (F) and novel (N) objects are indicated. **h** Quantification of exploration time for familiar and novel objects (left) and discrimination index (right) during the NOR test. All quantifications are presented as mean ± SD. Each dot represents one mouse (*n* = 25–30 mice per genotype). Escape latency during MWM training was analyzed using a mixed-effects model (REML), with training day as a repeated factor and genotype as a between-subject factor, followed by Sidak’s multiple comparisons test. Exploration time during the NOR test was analyzed using two-way ANOVA followed by Sidak’s multiple comparisons test. All other comparisons were analyzed using unpaired two-tailed Student’s t-test. **P* < 0.05; ns, not significant.

**Fig. S4** Impaired learning and memory in 3-MO *VPS35^CaMKIIa^* female mice. **a** Representative exploration heat maps from the NOR test in 3-MO female control and *Vps35^CaMKIIa^* mice. **b** Quantification of exploration time for familiar and novel objects (left) and discrimination index (right). All quantifications are presented as mean ± SD. Each dot represents one mouse (*n* = 10–15 mice per genotype). Exploration time was analyzed using two-way ANOVA with object (familiar vs. novel) and genotype as factors, followed by Sidak’s multiple comparisons test. Discrimination index was analyzed using unpaired two-tailed Student’s t-test. **P* < 0.05; ns, not significant.

**Fig. S5** Decreased Kir_2.1_ expression and function in hippocampal pyramidal neurons. **a** Representative voltage-gated sodium (Na^+^) channel currents recorded from CA1 pyramidal neurons under control conditions and in the disease model (left), together with quantification of peak sodium current density (right). **b** Representative inward rectifier potassium (Kir) channel currents recorded from CA1 pyramidal neurons (left) and corresponding current–voltage (I-V) relationships (right). **c** Representative Western blots showing the expression of Kir_2.1_ and retromer-associated proteins in hippocampus, cortex, and pons. **d-e** Quantification of Kir_2.1_ protein levels (**d**) and retromer complex components (VPS35, VPS26a, and VPS29) (**e**) in the hippocampus, cortex, and pons. **f** Representative immunofluorescence images showing Kir_2.1_ expression in CA1 pyramidal neurons. **g** Quantification of Kir_2.1_ fluorescence intensity in CaMKIIα-positive neurons. All quantifications are presented as mean ± SD. Each dot represents one neuron or one sample, as indicated. Peak voltage-gated sodium currents were analyzed using unpaired two-tailed Student’s t tests (*n* = 10-12 neurons per group, obtained from at least three mice per genotype). Inward K^+^ channel current–voltage relationships were analyzed using a mixed-effects model (REML), with test potential as a repeated factor and genotype as a between-subject factor, followed by Sidak’s multiple comparisons test (*n* = 10-12 neurons per group, obtained from at least four mice per genotype). Protein expression levels and fluorescence intensity were analyzed using two-way ANOVA with brain region and genotype as factors, followed by Sidak’s multiple comparisons test (*n* = 4-6 samples or 3-5 image fields per region per mouse, obtained from at least three mice per genotype). **P* < 0.05, ***P* < 0.01, ****P* < 0.001; ns, not significant.

**Fig. S6** Adult-onset hippocampal Vps35 deletion recapitulates CA1 neuronal hyperexcitability, E/I imbalance, and impaired recognition memory. **a** Experimental timeline and schematic illustration of adult-onset hippocampal Vps35 deletion. AAV-hSyn-GFP or AAV-hSyn-Cre was stereotaxically injected into the CA1 region of 2-MO Vps35^f/f^ mice, followed by electrophysiological recordings and behavioral analyses at 3-MO of age. **b-c** Representative voltage responses of CA1 pyramidal neurons evoked by 1-s depolarizing current injections during whole-cell current-clamp recordings (**b**), and firing frequency plotted against injected current intensity (**c**). **d-f** Resting membrane potential (RMP) (**d**), input resistance (**e**), and membrane capacitance (**f**) of CA1 pyramidal neurons recorded from control and adult-KO mice. **g-h** Representative traces of evoked excitatory postsynaptic currents (eEPSCs) and evoked inhibitory postsynaptic currents (eIPSCs) recorded from CA1 pyramidal neurons (**g**), and quantification of the evoked EPSC/IPSC ratio (**h**). **i-k** Novel object recognition (NOR) test. Representative exploration heat maps from the NOR test are shown in (**i**), quantifications of exploration time for familiar and novel objects (**j**) and discrimination index (**k**) are shown. All quantifications are presented as mean ± SD. Each dot represents one neuron or one mouse, as indicated. Action potential firing frequency was analyzed using a mixed-effects model (REML), with current step as a repeated factor and viral treatment as a between-subject factor, followed by Sidak’s multiple comparisons test (*n* = 9–12 neurons per group, obtained from at least four mice per group). Exploration time during the NOR test was analyzed using two-way ANOVA with object type and viral treatment as factors, followed by Sidak’s multiple comparisons test. Resting membrane potential, input resistance, membrane capacitance, evoked EPSC/IPSC ratio, and NOR discrimination index were analyzed using unpaired two-tailed Student’s t-test. **P* < 0.05; ***P* < 0.01; ns, not significant.

**Fig. S7** Restored Kir2.1 relieved seizure susceptibility and decreased CA1 neuronal hyper-excitability. **a** Schematic illustration of AAV-mediated Kir_2.1_ expression using a Syn-DIO-Myc-Kir_2.1_-mCherry construct. Viral injections were performed at 2 months of age, followed by electrophysiological and behavioral analyses at 3 months of age. **b** Representative coronal brain sections showing ipsilateral and contralateral expression of AAV-DIO-Kir_2.1_. Insets show higher-magnification images of Myc and mCherry signals. **c** Representative immunoblots showing Myc-tagged Kir_2.1_ expression in ipsilateral and contralateral hippocampi, with β-actin as a loading control. **d-f** Representative firing responses of CA1 pyramidal neurons recorded under current-clamp conditions following injection of control virus or Kir_2.1_-expressing virus and littermate controls (**d**), and quantification of the frequency-current relationships (**e**), and resting membrane potential (**f**). **g-h** Representative EEG traces recorded following viral expression of GFP or Kir_2.1_in *Vps35^CaMKIIa^* and control mice (**g**), and quantification of epileptic spike frequency over time (**h**). **i-j**. Representative eEPSC and eIPSC traces recorded from CA1 pyramidal neurons in control mice and *Vps35^CaMKIIa^* mice expressing vehicle or Kir_2.1_ (**i**), and quantification of the evoked EPSC/IPSC ratio (**j**). All quantifications are presented as mean ± SD. Each dot represents one neuron or mouse. Action potential firing frequency was analyzed using a mixed-effects model (REML), with current step as a repeated factor and group as a between-subject factor, followed by Sidak’s multiple comparisons test. Resting membrane potential was analyzed using one-way ANOVA followed by Tukey’s multiple comparisons test (*n* = 9–12 neurons per group, at least four mice per genotype). EEG signals were analyzed using or one-way ANOVA followed by Tukey’s multiple comparisons test (*n* = 8–9 mice per group). **P* < 0.05; ***P* < 0.01; ****P* < 0.001; ns, not significant.

**Fig. S8** Kir_2.1_ restoration was insufficient to rescue learning and memory in *Vps35^CaMKIIa^.* **a-b** Representative exploration heat maps from the novel NOR test following viral expression of GFP or Kir2.1in *Vps35^CaMKIIa^* and control mice (**a**), and quantification of exploration time for familiar (F) and novel (N) objects (**b**) and discrimination index (**c**). **d-f** Representative heat maps from the MWM probe trial following viral expression of GFP or Kir_2.1_ in *Vps35^CaMKIIa^* and control mice (**d**). The escape latency during MWM training across consecutive training days (**e**), and quantification of platform crossings during the MWM probe trial (**f**). **g-h** Representative heat maps from the OFT (**g**), and quantification of total distance traveled (left) and time spent in the center zone (right) during the OFT (**h**). All quantifications are presented as mean ± SD. Each dot represents one mouse. Exploration time in the novel object recognition (NOR) test was analyzed using two-way ANOVA with object (familiar vs. novel) and treatment as factors, followed by Sidak’s multiple comparisons test (*n* = 7–9 mice per group). MWM training curves were analyzed using a mixed-effects model (REML), with training day as a repeated factor and treatment as a between-subject factor, followed by Sidak’s multiple comparisons test (*n* = 20–24 mice per group). All others were analyzed using one-way ANOVA followed by Tukey’s multiple comparisons test. **P* < 0.05; ***P* < 0.01; ns, not significant. **i** Overview schematic illustrating the effects of Kir2.1 restoration on intrinsic excitability and behavioral phenotypes.

**Fig. S9** Levetiracetam attenuates intrinsic excitability of CA1 pyramidal neurons. **a** Representative action potential firing responses evoked by depolarizing current injections in CA1 pyramidal neurons under vehicle- or levetiracetam-treated conditions. **b-c** Frequency-current relationships of CA1 pyramidal neurons (**b**) and quantification of input resistance (left) and resting membrane potential (right) (**c**). **d** Quantification of hippocampal Aβ42 levels in the indicated groups. All quantifications are presented as mean ± SD. Each dot represents one neuron. Frequency–current relationships were analyzed using a mixed-effects model (REML), with current step as a repeated factor and genotype and treatment as between-subject factors, followed by Sidak’s multiple comparisons test (*n* = 9-12 neurons per group at least four mice per genotype). Input resistance, resting membrane potential, and Aβ42 levels were analyzed using one-way ANOVA followed by Sidak’s multiple comparisons test (*n* = 7–8 neurons per group at least three mice per genotype). **P* < 0.05; ***P* < 0.01; ****P* < 0.001; ns, not significant.

**Fig. S10** LEV rescues deficits in inhibitory synaptic transmission. **a** Representative traces of spontaneous inhibitory postsynaptic currents (sIPSCs) recorded from CA1 pyramidal neurons in 2-MO control and *Vps35^CaMKIIα^* mice. **b-c** Cumulative probability distributions of sIPSC inter-event intervals (**b**) and amplitudes (**c**), insets show quantifications respectively. **d** Representative paired-pulse inhibitory postsynaptic current (IPSC) traces recorded from CA1 pyramidal neurons in 2-MO control and *Vps35^CaMKIIα^* mice. **e** Quantification of paired-pulse ratio (PPR) at the indicated interstimulus intervals. **f** Representative paired-pulse IPSC traces recorded from CA1 pyramidal neurons in control mice and Vps35*^CaMKIIα^* mice treated with vehicle (Veh) or levetiracetam (LEV). **g**. Quantification of PPR at the indicated interstimulus intervals. LEV treatment did not significantly alter PPR in Vps35CaMKIIα mice. All quantifications are presented as mean ± SD. Each dot represents one neuron. Cumulative distributions of sIPSC inter-event intervals and amplitudes were analyzed using the Kolmogorov–Smirnov test. Mean sIPSC frequency and amplitude were analyzed using unpaired two-tailed Student’s t-test or one-way ANOVA followed by Tukey’s multiple comparisons test, as appropriate. PPR data were analyzed using a mixed-effects model (REML), with interstimulus interval as a repeated factor and genotype or treatment as a between-subject factor, followed by Sidak’s multiple comparisons test. **P* < 0.05; ***P* < 0.01; ns, not significant.

**Fig. S11** LEV rescues deficits in miniature inhibitory synaptic transmission. **a** Representative mIPSC traces from CA1 pyramidal neurons in 2-MO control and *Vps35^CaMKIIα^* mice. **b-c** Cumulative probability distributions of mIPSC inter-event intervals (**b**) and amplitudes (**c**), insets show quantifications respectively. **d** Representative mIPSC traces from CA1 pyramidal neurons in 3-MO control mice and *Vps35^CaMKIIα^* mice treated with vehicle (Veh) or levetiracetam (LEV). **e-f.** Cumulative probability distributions of mIPSC inter-event intervals (**e**) and amplitudes (**f**), insets show quantifications respectively. All quantifications are presented as mean ± SD. Each dot represents one neuron. Cumulative distributions of mIPSC inter-event intervals and amplitudes were analyzed using the Kolmogorov–Smirnov test. Mean mIPSC frequency and amplitude were analyzed using unpaired two-tailed Student’s *t*-test or one-way ANOVA followed by Tukey’s multiple comparisons test. **P* < 0.05; ***P* < 0.01; ns, not significant.

**Fig. S12** Age-dependent excitatory synapse impairment in the CA1 hippocampus of Vps35*^CaMKIIα^* mice**. a** Representative maximum intensity projection images showing immunostaining for PSD95 (green) and VGLUT1 (red) in the stratum radiatum (SR) of the CA1 region. **b** Quantification of PSD95 puncta size, PSD95 puncta density, VGLUT1 puncta density, and PSD95–VGLUT1 colocalized puncta density within defined regions of interest. **c** Representative immunoblots of PSD95 from hippocampal homogenates at 2 and 3 months of age under control, vehicle-treated, and levetiracetam-treated conditions, with β-actin as a loading control, and corresponding quantification of relative PSD95 protein levels. All quantifications are presented as mean ± SD. Each dot represents one image field or one biological sample, as indicated. Fluorescence intensities of PSD95, Vglut1, and PSD95/Vglut1 colocalization were analyzed using two-way ANOVA with genotype and treatment as factors, followed by Sidak’s multiple comparisons test (*n* = 6–8 fields per mouse, obtained from at least four mice per group). Protein expression levels in hippocampal homogenates were analyzed using one-way ANOVA followed by Sidak’s multiple comparisons test (*n* = 4–6 biological samples per group). **P* < 0.05; ***P* < 0.01; ns, not significant.
